# *In vitro*, *in vivo*, and *in silico* approaches for evaluating the preclinical DMPK profiles of ammoxetine, a novel chiral serotonin and norepinephrine reuptake inhibitor

**DOI:** 10.3389/fphar.2024.1486856

**Published:** 2024-11-07

**Authors:** Xiuqing Zhu, Yuexin Li, Huan Luo, Yunxia Zhang, Zhenqing Zhang, Jinglai Li

**Affiliations:** ^1^ Department of Pharmacy, The Affiliated Brain Hospital, Guangzhou Medical University, Guangzhou, China; ^2^ Key Laboratory of Neurogenetics and Channelopathies of Guangdong Province and the Ministry of Education of China, Guangzhou Medical University, Guangzhou, China; ^3^ Guollence Pharmaceutical Technology Co., Ltd., Beijing, China; ^4^ The Key Laboratory of Drug Metabolism and Pharmacokinetics, Beijing Institute of Pharmacology and Toxicology, Beijing, China

**Keywords:** ammoxetine, depression, antidepressant, serotonin and norepinephrine reuptake inhibitor, drug metabolism and pharmacokinetics, ADME, preclinical pharmacokinetics

## Abstract

**Background and Aim:**

Ammoxetine, a novel chiral serotonin and norepinephrine reuptake inhibitor, holds promise for major depressive disorder treatment. This study aimed to thoroughly investigate its preclinical drug metabolism and pharmacokinetics (DMPK) profiles.

**Methods:**

The preclinical DMPK profiles of ammoxetine were examined through *in vitro*, *in vivo*, and *in silico* methods.

**Results:**

Assessment of blood-brain barrier penetration *via* MDCK-MDR1 cells revealed strong brain permeation by ammoxetine, despite being a probable P-glycoprotein (P-gp) substrate. Molecular docking indicated a robust binding interaction between ammoxetine and P-gp. Ammoxetine was well absorbed orally, with T_max_ ranging from 0.75 to 3.83 h in rats and 0.75–1.40 h in beagle dogs. At a 2 mg/kg dose in beagle dogs, ammoxetine exhibited an absolute bioavailability of approximately 42%. Plasma protein binding rates were around 50%–60% in beagle dogs, rats, and humans, suggesting moderate binding. Tissue distribution studies displayed rapid and extensive ammoxetine spread in major rat tissues post-gavage, with notable brain exposure and no tissue accumulation. Cumulative excretion rates in rats’ urine, feces, and bile accounted for only 1.11% of the total administered drug, indicating extensive transformation into metabolites. Chiral inversion of ammoxetine was absent *in vivo*. Metabolic stability varied across species using liver microsomes, but beagle dogs showed clearance rates more akin to humans. Metabolic pathways unveiled two key metabolites, M1 and M2. M1, likely generated through methylenedioxyphenyl ring oxidation, involves CYP2C19 and CYP3A4, crucial human cytochrome P450 (CYP) enzymes for liver metabolism, while M2 is M1’s glucuronide conjugate. Ammoxetine may exhibit saturation elimination trends with increasing doses in rats and beagle dogs. A high-throughput assay using the cocktail-substrate method indicated weak CYP inhibition by ammoxetine on CYP2D6 and CYP1A2, with minimal effects on other CYP enzymes, suggesting a low likelihood of CYP inhibition-related drug-drug interactions.

**Conclusion:**

This study presents encouraging DMPK profiles of ammoxetine, backing its potential as a candidate compound for future clinical assessments.

## Introduction

Major depressive disorder (MDD) is a prevalent mental disorder characterized by persistent depressed mood and diminished interests. It significantly impairs psychosocial functioning, diminishes the quality of life in affected individuals, and is projected to become the leading global disease burden by 2030 (Malhi and Mann, 2018). The monoamine hypothesis serves as the foundation for the development and utilization of most currently prescribed antidepressants, which aim to restore depleted levels of monoamine neurotransmitters, such as serotonin (5-HT) and norepinephrine (NE) (Pastis et al., 2024). Selective serotonin and norepinephrine reuptake inhibitors (SNRIs) represent a prominent class of newer-generation antidepressants that act by simultaneously inhibiting the reuptake of 5-HT and NE through targeted binding to their respective transporters, thereby elevating their levels within the synaptic cleft of the brain. Recent meta-analyses have indicated that SNRIs, including venlafaxine and duloxetine, exhibit superior efficacy or tolerability compared to other antidepressants in the treatment of MDD (Kishi et al., 2023; Yuan et al., 2020). However, despite being well-tolerated, duloxetine may induce several common adverse effects, such as nausea, somnolence, dry mouth, sweating, constipation, and decreased appetite, in depressed patients. Furthermore, cases of duloxetine-associated hepatotoxicity have been reported in recent years, imposing limitations on its clinical use, particularly in patients with significant liver disease (Jiang et al., 2024). Nevertheless, continuous efforts are underway to develop new antidepressants to address the escalating burden of depression.

**FIGURE 1:**
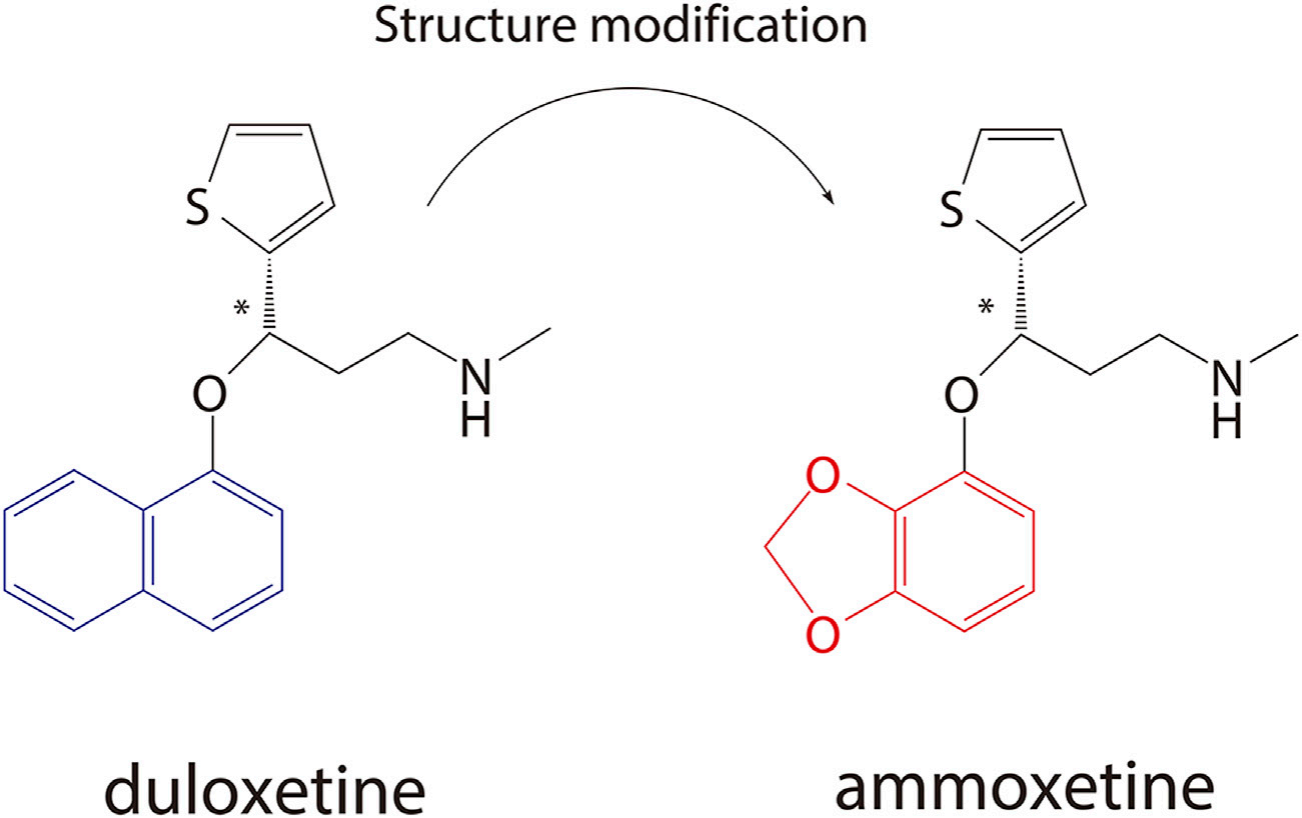
Chemical structures of duloxetine and ammoxetine.

Ammoxetine, currently undergoing phase II clinical trials in China (registered at chinadrugtrials.org.cn/index.html, registration number: CTR20211677; clinicaltrials.gov, identifier: NCT05018013), represents a novel and potent balanced SNRI compound derived from duloxetine (Figure 1) (Xue et al., 2013b). It is worth noting that ammoxetine is a chiral compound, specifically the S-(−) isomer of 071031B ((±)-3-(benzo[d] [1,3]dioxol-4-yloxy)-N-methyl-3-(thiophen-2-yl)propan-1-amine) (S-071031B). Previous studies have revealed that duloxetine-induced liver injuries are associated with its potential toxicophore, the naphthyl ring, which triggers oxidative stress and leads to mitochondrial dysfunction in hepatic organoids. Notably, the structural modification of ammoxetine, replacing the naphthyl ring with benzodioxole, contributes to a reduction in duloxetine-induced hepatotoxicity (Liu et al., 2022). Furthermore, animal models of depression have demonstrated that ammoxetine exhibits superior antidepressant effects along with lower hepatotoxicity and neurotoxicity compared to duloxetine (Xue et al., 2013a). Additionally, in animal models of inflammatory and continuous pain, ammoxetine has shown more potent analgesic effects than duloxetine (Zhang et al., 2016). Similar to duloxetine, ammoxetine can alleviate mechanical allodynia in diabetic rats over a chronic period without affecting their blood glucose levels or body weight (Zhang et al., 2018). Based on these findings, it is evident that ammoxetine holds potential clinical value comparable to duloxetine while offering a potentially safer alternative for the treatment of depression.

Fortunately, despite the instability of ammoxetine and its R-enantiomer in citric acid/phosphate buffer (pH 2) (Xue et al., 2017), significant progress has been made in the development of ammoxetine hydrochloride enteric-coated tablets. A first-in-human study has assessed the safety, tolerability, and pharmacokinetics (PK) of ammoxetine in healthy Chinese subjects, revealing favorable PK profiles following oral administration and demonstrating good safety properties (Shen et al., 2021). Chiral compounds often exhibit distinct properties, displaying stereoselectivity in their PK and pharmacodynamics (PD). For instance, a previous chiral analysis of ammoxetine enantiomers suggested potential stereoselectivity in the PK of ammoxetine enantiomers in dogs (Yu et al., 2017). Although a previous pre-clinical study has highlighted the divergent PK profiles, rather than pH stability, of ammoxetine and its R-enantiomer, contributing to their differential behavioral pharmacology (Xue et al., 2017), the comprehensive and systematic assessment of the preclinical drug metabolism and pharmacokinetics (DMPK) profiles of ammoxetine *in vitro* and *in vivo* remains undisclosed.

Therefore, the objectives of the current study are as follows: 1) investigate the mechanisms of brain penetration of ammoxetine, specifically focusing on the P-glycoprotein (P-gp, MDR1)-mediated efflux at the blood-brain barrier (BBB). This will be accomplished through the use of *in vitro* assays using Madin-Darby canine kidney cells transfected with the human *MDR1* gene (MDCK-MDR1). Additionally, potential binding interactions between ammoxetine and human P-gp will be explored using molecular docking methods; 2) compare the species differences in the metabolic stability of ammoxetine in liver microsomes and elucidate its main metabolic pathways using both *in vitro* and *in vivo* approaches; 3) assess whether ammoxetine acts as a substrate or inhibitor of human cytochrome P450 (CYP) enzymes; and 4) characterize the *in vivo* configurational stability of ammoxetine, along with its preclinical PK, tissue distribution, plasma protein binding (PPB), and excretion profiles in different species. These comprehensive studies will provide valuable insights for the future clinical development of ammoxetine and aid in decision-making regarding drug-drug interactions.

## Materials and methods

### Materials

Ammoxetine and its R-enantiomer (both with purities above 99.0%), as well as L-phencynonate hydrochloride [internal standard (IS), purity above 99.0%], were synthesized at the Beijing Institute of Pharmacology and Toxicology (Beijing, China). High-performance liquid chromatography (HPLC) grade methanol, acetonitrile, n-hexane, and diethylamine were purchased from Sigma-Aldrich (St. Louis, USA), while isopropanol was obtained from Anhui Fulltime Specialized Solvent & Reagent Co., Ltd. (Anqing, China). Verapamil was supplied by Honeywell-Fluka (Charlotte, USA), and Dexmedetomidine (IS) was provided by Dexinjia Biopharm Co., Ltd. (Jinan, China). Nicotinamide adenine dinucleotide phosphate (NADPH) was obtained from Roche (Mannheim, Germany). Blank healthy plasma was supplied by the 307 Hospital (Beijing, China). Solid-phase extraction (SPE) cartridges (Oasis@HLB 1cc) were purchased from Waters (Milford, USA), and ultrafiltration devices (Amicon^®^ Ultra-0.5) were supplied by Merck Millipore (Burlington, USA).

MDCK-MDR1 cells were provided by Prof. Su Zeng (College of Pharmaceutical Sciences, Zhejiang University, Hangzhou, China). Dulbecco’s modified Eagle’s medium (DMEM) was purchased from Gibco (Waltham, USA). Fetal bovine serum (FBS) and non-essential amino acids (NEAA) were obtained from Hyclone (Logan, USA). 4-(2-hydroxyethyl) piperazine-1-ethanesulfonic acid (HEPES) and trypsin (1:250) were supplied by Amresco (Solon, USA). Glutamine was provided by Sigma-Aldrich (St. Louis, USA). Penicillin and streptomycin were purchased from Huabei Pharmaceutical Co., Ltd. (Shijiazhuang, China). Thiazolyl blue tetrazolium bromide powder (MTT) and ethylenediaminetetraacetic acid (EDTA)-Na_2_ were obtained from Beijing Chemical Works (Beijing, China).

Pooled liver microsomes of rat (RLM), dog (DLM), monkey (MLM), and human (HLM), as well as recombinant human CYP enzymes including CYP 1A2, CYP 3A4, CYP 2C9, CYP 2C19, and CYP 2D6, were purchased from BD (Woburn, USA). The CYP-specific probe substrates, phenacetin, coumarin, tolbutamide, and chlorozoxazone, were obtained from Sigma-Aldrich (St. Louis, USA). Bupropion and S-mephenytoin were purchased from Santa Cruz Biotechnology (Santa Cruz, USA), dextromethorphan from Tocris Bioscience (Bristol, United Kingdom), amodiaquine from Honeywell-Fluka (Charlotte, USA), testosterone from Perfemiker (Shanghai, China), and midazolam from Iphase Biosciences (Beijing, China). Metabolites of the CYP probe substrates, including acetaminophen, 4-hydroxytolbutamide, 4-hydroxy mephenytoin, and 6β-hydroxytestosterone, were purchased from Sigma-Aldrich (St. Louis, USA). 7-hydroxycoumarin, hydroxybupropion, and 6-hydroxychlorzoxazone were obtained from BD (Woburn, USA), desethylamodiaquine from Toronto Research Chemicals (Toronto, Canada), 1′-hydroxymidazolam from Cayman Chemical Company (Ann Arbor, USA), and dextrorphan from Perfemiker (Shanghai, China). All other chemicals were of analytical grade and used as received.

### Animals

Sprague-Dawley (SD) rats weighing (200 ± 20) g (male) and Beagle dogs weighing (10 ± 2) kg (male and female) were obtained from the Academy of Military Medical Sciences Animals Center (Beijing, China, permit number SCXK(JUN) 2007-004) and Beijing Marshall Biotechnology Co., Ltd. (Beijing, China, permit number SCXK(JING) 2009-0002), respectively. All animals were individually housed in stainless-steel cages at a controlled temperature of (25 ± 2)°C and provided with *ad libitum* access to food and water. The animal experiments were performed in compliance with the requirements of the National Institutes of Health (NIH) guide for the care and use of laboratory animals (NIH publication No. 86–23, revised 1996) and the guidelines approved by the Academy of Military Medical Sciences for ensuring the welfare and ethical treatment of animals used for scientific purposes in the course of the study.

### Instrumentation

For the determination of concentrations of ammoxetine and identification of its major metabolites, a Finnigan TSQ Quantum liquid chromatography-tandem mass spectrometry (LC-MS-MS) system (Thermo Fisher Scientific, Waltham, USA) and a Thermo Fisher C_18_ column were utilized. Metabolites from CYP probe substrates were analyzed using an AB Sciex API 5000 LC-MS-MS system (Foster City, USA) equipped with a Shimadzu LC-20AD HPLC system (Kyoto, Japan) and a Thermo Fisher C_18_ column. The analysis of *in vivo* chiral inversion of ammoxetine was performed on an Agilent 1100 LC/MSD VL LC-MS system (Santa Clara, USA) using a CHIRALPAK^®^ AD-H column supplied by Daicel Chemical Industries, LTD (Tokyo, Japan).

### MDCK-MDR1 transport study

#### Cell culture

MDCK-MDR1 cells at passage 17 were cultured in DMEM supplemented with 10% FBS, 1% glutamine, 100 U/mL penicillin/streptomycin, 1% NEAA, and 1 mmol/L sodium pyruvate. The cells were incubated overnight at 37°C with 5% CO_2_ and a relative humidity of 90%. Once the cells reached approximately 80% confluence, they were treated with a 0.25% trypsin/EDTA solution to dissociate the cells. Subsequently, the cells were seeded at a density of 5 × 10^5^ cells/cm^2^ in Millicell-96 well plates (Millipore, Burlington, USA). Prior to conducting the experiments, the integrity of the cell monolayer was confirmed by measuring transepithelial electrical resistance (TEER) using a Millicell ERS^®^ meter (Millipore, Burlington, USA). A TEER value greater than 400 Ω cm^2^ was required to ensure the formation and preservation of tight junctions.

#### Cytotoxicity assays

The ability of ammoxetine to inhibit the growth of MDCK-MDR1 cells was evaluated using the MTT assay. Ammoxetine was diluted with transport buffer [Hank’s balanced salt solution (HBSS) containing 6.0 g HEPES, pH 7.2–7.4] from a stock solution (2 mg/mL) dissolved in HBSS to create a series of standard concentrations: 1.28, 6.4, 32, 160, 800 ng/mL and 4, 20, 100, 500 μg/mL. Prior to the assay, the cell culture medium was aspirated and replaced with different standard concentrations of ammoxetine (100 μL/well, n = 6). The cells were then incubated at 37°C for 3 h. Following the incubation, 20 μL of MTT solution (5 mg/mL) was added to each well, and the incubation was continued for 4 h. Subsequently, the solution in each well was discarded, and 150 μL of dimethyl sulfoxide was added. After vortexing for 10 min, the absorbance was measured at 570 nm using a Varioskan^®^ Flash multimode reader (Thermo Fisher Scientific, Waltham, USA). Blank control solutions, consisting of HBSS without ammoxetine, were also applied to the cells. The absorbance values were then used to calculate the relative growth rate (RGR, %) of cells using the formula RGR = OD_ammoxetine_/OD_blank_ × 100%, where OD_ammoxetine_ and OD_blank_ represent the mean absorbance values of six parallel measurements in the ammoxetine and blank control solutions, respectively (Ji et al., 2024). The Prism 5 software (GraphPad Software, Inc., San Diego, USA) was used to determine the 50% toxic concentration (TC_50_) value of ammoxetine.

#### Bidirectional permeability measurements

Cell monolayers were preincubated with HBSS for 15 min at 37°C. To measure the A→B (apical side to basolateral side of monolayer) transporter, 400 μL of ammoxetine solution was added to the A-side (donor chamber), and 1,200 μL of HBSS solution was added to the B-side (receiver chamber). The B→A transporter was evaluated by adding 1,200 μL of ammoxetine solution to the B-side (donor chamber) and 400 μL of HBSS solution to the A-side (receiver chamber). At 0 h, 50 μL of the solution was taken from the donor chamber, and aliquots of 50 μL were taken from the receiver chamber at 15, 30, 45, 60, 90, and 120-min time intervals. The collected aliquots were immediately replaced with equal volumes of fresh blank HBSS. Ammoxetine transport was studied at three concentrations: 0.2, 1, and 5 μg/mL, with each concentration evaluated in triplicate. Additionally, the P-gp inhibitor verapamil at a concentration of 100 μmol/L was used to assess the altered transport of ammoxetine at concentrations of 0.2, 1, and 5 μg/mL. Ammoxetine samples collected from the receiver chambers at different time points were mixed with 150 μL of acetonitrile/methanol (1:1, *v*/*v*) solution containing 50 ng/mL L-phencynonate (IS). The mixtures were then vortexed for 1 min and centrifuged for 10 min at 13,800×*g*. The supernatants were analyzed using an LC-MS-MS method.

The apparent permeability coefficient ( $P_{app}$ , cm/s) values for ammoxetine were determined using Equation 1:

$$P_{app}=\left(dQ/dt\right)/\left(A\times C_{0}\right)$$ (1)

Here, dQ/dt represents the amount of drug transported to the receiver chamber within a specific time period, A is the surface area of the cell monolayer (=0.33 cm^2^), and $C_{0}$ is the initial concentration of ammoxetine.

In general, the involvement of a P-gp-mediated efflux mechanism was indicated if the efflux ratio ( $E_{R}$ ) value exceeded 1.5 (Langthaler et al., 2024). $E_{R}$ was calculated using Equation 2:

$$E_{R}=P_{app\left(B\to A\right)}/P_{app\left(A\to B\right)}$$ (2)

#### Molecular docking between ammoxetine and P-gp

The high-resolution 3D structure of the P-gp protein (6C0V) was obtained from the Protein Data Bank (PDB) website (www.rcsb.org) by configuring the species with “*Homo sapiens*.” The structure was prepared using AutoDockTools version 1.5.7 from The Scripps Research Institute Molecular Graphics Laboratory in La Jolla, United States of America. The preparation process involved removing water molecules, adding charge, hydrogenation, and other necessary operations. The resulting structure was saved as a “PDBQT” file. The structure of ammoxetine, generated using ChemDraw, was converted to the “MOL2” format using Chem 3D 20.0 software. Subsequently, it was imported into AutoDockTools to determine the rotatable bonds and saved as a “PDBQT” file. Molecular docking and analysis of protein-ligand interactions were performed using AutoDockVina, PyMOL 2.3.0, and the online tool Protein-Ligand Interaction Profiler (PLIP) (https://plip-tool.biotec.tu-dresden.de/plip-web/plip/index). Binding energy values lower than −7 kcal/mol indicate a very strong binding activity, while values lower than −5 kcal/mol indicate strong binding activity.

### Metabolic disposition study

#### Hepatic microsome stability assay

A hepatic microsome stability assay was conducted to investigate the metabolic disposition of ammoxetine. In this assay, 100 μL of ammoxetine (2.5 μmol/L) was incubated at 37°C with 100 μL of liver microsomes (RLM, DLM, MLM, or HLM) formulated in a 5 mmol/L dipotassium hydrogen phosphate (K_2_HPO_4_) buffer at pH 7.4. The concentration of microsomes was 0.5 g/L. To initiate the incubations, 50 μL of NADPH solution preincubated at 37°C was added. This resulted in final concentrations of 1 μmol/L for ammoxetine, 0.2 g/L for liver microsomes, and 1 mmol/L for NADPH in the reaction system. Parallel triplicate samples were prepared for each time point, including 0, 2, 5, 10, 15, 30, 60, 90, and 120 min. Parallel triplicate samples were prepared for each time point, including 0, 2, 5, 10, 15, 30, 60, 90, and 120 min. To stop the reactions at each time point, 250 μL of ice-cold precipitating agent (acetonitrile/methanol, 1:1, *v*/*v*) containing 500 ng/mL of L-phencynonate (IS) was added to the incubation mixture. The samples were vortexed for 2 min at 4°C and then centrifuged at 13,800×*g* for 10 min. The supernatants were analyzed using an LC-MS-MS approach. To ensure the reliability of the assay, a negative control without NADPH and a positive control with verapamil (final concentration 1 μmol/L) were conducted alongside the experimental groups.

The calculation of intrinsic clearance (*CL*_*int*_) and predicted hepatic clearance (*CL*_*h*_) in this study is based on a method described in the literature (Liu et al., 2014). Formulas 3, 4 are used to determine these parameters:

$${CL}_{\mathrm{int}}=\frac{0.693}{t_{1/2}}\times \frac{Incubation\left(mL\right)}{Microsomes\left(mg\right)}\times \frac{Microsomes\left(mg\right)}{Liver\left(g\right)}\times \frac{Liver\left(g\right)}{BW\left(kg\right)}$$ (3)

$${CL}_{h}=\frac{Q_{h}\times {CL}_{\mathrm{int}}}{Q_{h}+{CL}_{\mathrm{int}}}$$ (4)

In Formula 3, $t_{1/2}$ (min) represents the half-life of ammoxetine in liver microsomes. It is calculated by dividing −0.693 by the resulting slope (*k*_e_) obtained from a natural logarithm plot of the remaining concentration of ammoxetine against incubation time. The value of $\frac{Microsomes\left(mg\right)}{Liver\left(g\right)}$ is 45 for all species, while $\frac{Liver\left(g\right)}{BW\left(kg\right)}$ is 40, 32, 30, and 25.7 for rats, dogs, monkeys, and humans, respectively. The liver blood flow ( $Q_{h}$ ) is 55.2, 30.9, 43.6, and 20.7 mL/min/kg for the respective species (Davies and Morris, 1993).

#### Initial identification of major metabolites

For the initial identification of major metabolites, triplicate samples of ammoxetine were subjected to *in vitro* metabolite profiling and characterization. The samples were incubated in RLM, DLM, MLM, and HLM in a 5 mmol/L K_2_HPO_4_ buffer at pH 7.4, at a temperature of 37°C for 2 h. NADPH was present during the incubation process. This resulted in a total volume of 250 μL of incubation mixture with final concentrations of 20 μmol/L for ammoxetine, 0.4 g/L for liver microsomes, and 1 mmol/L for NADPH. To stop the reactions and extract the metabolites, equal volumes of ice-cold precipitating agents (acetonitrile/methanol, 1:1, *v*/*v*) were added to the incubation mixture. The samples were then vortexed for 2 min at 4°C and centrifuged at 13,800×*g* for 10 min. The supernatants obtained were subjected to analysis using an LC-MS-MS approach (Li et al., 2006). To ensure the reliability of the assay, a blank control without ammoxetine and a positive control with verapamil were performed in parallel with the experimental samples.

*In vivo* assessment of metabolites was also conducted using urine samples from SD rats. Three SD rats were individually housed in metabolic cages with access to water and a solid diet for 2 days to allow acclimation. Following this, the rats underwent a 24-h fasting period, during which blank urine samples were collected as controls. Each rat received an oral dose of 50 mg/kg of ammoxetine at 3-h intervals for a total of four doses. Urine samples were collected from the rats at 24 h post-dose and stored at approximately −30°C until sample preparation using the SPE method. After thawing, 2 mL aliquots of urine samples were loaded onto SPE cartridges that had been conditioned with 1 mL of methanol and 1 mL of water. The cartridges were then washed with 1 mL of water and eluted with 2 mL of methanol. The eluate was evaporated to dryness under nitrogen at 40°C, and the residues were reconstituted in 100 μL of methanol for LC-MS-MS analysis (Li et al., 2006). To provide a mechanistic understanding of fragmentation schemes and confirmatory MS/MS metabolite structural determination, Mass Frontier 4.0 (Thermo Fisher Scientific, Waltham, USA) was utilized.

### CYP phenotyping and inhibition study

#### Phenotyping of recombinant human CYP isoenzyme

The incubation system had a final volume of 200 μL. Ammoxetine (final concentration 5 μmol/L) was incubated with recombinant human CYP 3A4, CYP 1A2, CYP 2C9, CYP 2C19, and CYP 2D6 (final concentration 25 pmol/mL) in a 5 mmol/L K_2_HPO_4_ buffer at pH 7.4. The incubations were performed in quintuplicate. NADPH (final concentration 1 mmol/L) was added to initiate the incubation process. The mixture was then incubated at 37°C for 1 h. To stop the reaction, 200 μL of ice-cold acetonitrile/methanol (1:1, *v*/*v*) containing 500 ng/mL of L-phencynonate (IS) was added. The samples were vortexed for 2 min and centrifuged at 13,800×*g* and 4°C for 10 min. The supernatants obtained were analyzed using an LC-MS-MS method. To establish appropriate controls, zero-time controls and blank controls in the absence of NADPH were set up in parallel.

#### CYP inhibition experiment

To assess the CYP inhibition profiling of ammoxetine, a high-throughput assay based on the cocktail-substrate method was performed, as described in the literature (Kim et al., 2005; Kozakai et al., 2014). Microsomal incubations were conducted in triplicate for 30 min at 37°C in a reaction system (200 μL) consisting of 0.1 mmol/L potassium phosphate buffer (pH 7.4), 0.5 mg/mL HLM, 1 mmol/L NADPH, and a cocktail of 10 probe substrates for 9 CYP isoforms. The final concentration of each CYP-specific substrate was chosen to be close to the Michaelis-Menten constant (*K*_*m*_) value of the respective substrate (Kim et al., 2005; Lee et al., 2024). For instance, phenacetin (CYP 1A2) was used at a concentration of 50 μmol/L, coumarin (CYP 2A6) at 5 μmol/L, bupropion (CYP 2B6) at 50 μmol/L, amodiaquine (CYP 2C8) at 5 μmol/L, tolbutamide (CYP 2C9) at 100 μmol/L, S-mephenytoin (CYP 2C19) at 100 μmol/L, dextromethorphan (CYP 2D6) at 5 μmol/L, chlorzoxazone (CYP 2E1) at 50 μmol/L, and testosterone and midazolam (CYP 3A4) at 50 μmol/L. Ammoxetine was evaluated at five concentrations: 0.1, 1, 10, 50, and 100 μmol/L. Additionally, a blank control without ammoxetine was included. The incubation was terminated by adding an equal volume of ice-cold acetonitrile/methanol (1:1, *v*/*v*) along with the IS, dexmedetomidine (1 ng/mL). After a 20-fold dilution, the mixture was centrifuged at 13,800×*g* for 10 min at 4°C. The supernatants were used to measure the levels of metabolites from the respective probe substrates using an LC-MS-MS method. The half-maximal inhibitory concentration (IC_50_) values were subsequently calculated.

### PPB assay

The PPB of ammoxetine was determined using the ultrafiltration method. Triplicate samples of rat, dog, or human plasma were spiked with ammoxetine at three final concentrations (50, 150, and 450 ng/mL). The spiked plasma samples were then incubated in a 37°C air-bath shaker for 1 h until equilibrium was reached. After incubation, 400 μL of the plasma samples were transferred to pre-saturated ultrafiltration devices. These devices were subsequently centrifuged at 13,800×*g* and 20°C for 20 min to separate the unbound ammoxetine. 100 μL of the test samples were taken and mixed with 100 μL of the IS, L-phencynonate (50 ng/mL), and 300 μL of methanol. Following centrifugation at 13,800×*g* for 10 min, the supernatants were used to determine the total and free concentrations of ammoxetine in the plasma (*C*_*t*_, *C*_*f*_) using an LC-MS-MS method. The PPB rate (%) of ammoxetine was calculated by dividing (*C*_*t*_−*C*_*f*_) by *C*_*t*_.

### *In vivo* study

#### Configurational stability study

To demonstrate that chiral inversion of ammoxetine did not occur *in vivo*, an HPLC method utilizing a chiral stationary phase was developed for the separation and identification of enantiomers. Twelve SD rats were divided into three groups: a blank control group, an S-071031B group (50 mg/kg), and an R-071031B group (50 mg/kg). Prior to administration, the animals underwent a 12-h fasting period while having access to water. At 2 h post-dosing, the rats were anesthetized, and blood samples were collected from the abdominal aorta into heparinized tubes. Plasma samples were obtained by centrifuging the collected blood at 2,000×*g* for 10 min to remove blood cells. Subsequently, 1 mL of plasma sample was mixed with 100 μL of 0.1% sodium hydroxide solution and 4 mL of ether. After centrifugation at 2,000×*g* for 10 min, the upper organic phase within each group was combined, transferred to a new tube, and dried under a stream of nitrogen. The resulting residues were reconstituted with 100 μL of methanol for subsequent HPLC analysis.

#### PK studies in rats and dogs

For the PK studies in rats and dogs, a total of 18 SD rats and 15 male beagle dogs were randomly divided into three groups, with six rats or five dogs per group, respectively. Prior to the experiments, all animals underwent a 12-h fasting period, while having free access to water. The animals were administered single oral doses of ammoxetine in three different dose levels: 8 mg/kg, 20 mg/kg, and 50 mg/kg for rats, and 2 mg/kg, 5 mg/kg, and 12.5 mg/kg for dogs, representing low, medium, and high doses. Serial blood samples were collected from the rats at 0, 2, 5, 15, and 30 min, as well as 1, 2, 4, 6, 8, 12, and 24 h post-dosing. For the dogs, blood samples were collected at 0, 5, 15, 30, and 45 min, as well as 1, 2, 4, 6, 8, 12, and 24 h post-dosing. Approximately 0.3 mL of blood was collected from the rats, while 2 mL of blood was collected from the dogs. The blood samples were collected into heparinized tubes. Following centrifugation, plasma samples were obtained and stored at −30°C until further analysis.

For the absolute bioavailability (*F*, %) study in dogs, the dogs initially received a single gastric gavage of a low dose (2 mg/kg) of ammoxetine, followed by a 1-week washout period. After the washout, the same dose of ammoxetine was administered intravenously (i.v.). To assess the effects of nonfasting on ammoxetine PK, dogs that received a medium dose (5 mg/kg) of ammoxetine *via* gastric gavage underwent a radical washout period of 1 week. Subsequently, they were administered the same dose of ammoxetine *via* gastric gavage under nonfasting conditions. To compare sex differences in ammoxetine PK, an additional five female beagle dogs were administered a medium dose (5 mg/kg) of ammoxetine *via* gastric gavage after a 12-h overnight fast. Blood samples (2 mL) were collected from these dogs into heparinized tubes at predose and at specified time points: 5, 15, 30, and 45 min, as well as 1, 2, 4, 6, 8, 12, and 24 h postdose. For the repeat-dose study, after completing blood collection during the initial administration, dogs receiving a single gastric gavage of a medium dose (5 mg/kg) of ammoxetine under fasting conditions were subsequently orally dosed with seven repeat doses of ammoxetine (5 mg/kg) every 12 h. Trough blood samples were collected before the fourth to last administration to ensure steady-state blood concentrations (*C*_*ss*_) were achieved. Approximately 2 mL of blood was collected at specified time points after the last dose and transferred into heparinized tubes. All blood samples were then centrifuged at 4,000 r/min for 20 min to obtain plasma samples, which were stored at −30°C until analysis.

To determine the concentration of ammoxetine, 100 μL of L-phencynonate (IS, 50 ng/mL) and 300 μL of methanol were added to 100 μL of plasma samples from rats or dogs. After thorough vortexing, the mixture was centrifuged at 13,800×*g* for 10 min. The supernatants were then subjected to analysis using LC-MS-MS methods. PK parameters were calculated using DAS (Drug And Statistics for Windows) version 2.0, developed by the Shanghai University of Traditional Chinese Medicine, Shanghai, China. The *F* was determined by comparing the plasma area under the concentration-time curve (AUC) values after oral (p.o.) and intravenous (i.v.) administration of ammoxetine. *F* was calculated using the formula: $F=\left({AUC}_{p.o.}/{AUC}_{i.v.}\right)\times 100\%$ . Statistical analyses were performed using OriginPro 8.6 software. Paired sample or student’s t-test, as well as one-way ANOVA, were employed to compare the means of two or three groups, with a significance level set at *p* < 0.05.

#### Tissue distribution study in rats

A total of 24 rats were divided equally into four groups, with each group receiving a single oral gavage dose of 20 mg/kg of ammoxetine. Based on the rat PK analysis results for the 20 mg/kg dose, specific time points were selected for sampling, including before, at, and after the peak of ammoxetine plasma concentration (i.e., 0.25, 2, 6, and 24 h, respectively). At each designated time point after drug administration, the rats were euthanized, and approximately 1 mL of blood samples were collected into heparinized tubes. The heart, liver, spleen, lung, kidney, brain, intestine, stomach, testis, fat, and muscle tissues were rapidly removed after washing out the blood with ice-cold saline. The blood samples were then centrifuged at 6,000 r/min for 15 min to obtain plasma. The collected tissue samples were weighed and homogenized in distilled water to create a 10% homogenate. For further analysis, 100 μL of plasma or tissue homogenate samples were mixed with 300 μL of methanol and 100 μL of L-phencynonate (IS, 50 ng/mL). The mixture was vortexed for 1 min and then centrifuged at 13,800×*g* for 10 min. The resulting supernatants were used for LC-MS-MS analysis. Using the trapezoidal method, the tissue-to-blood AUC ratio ( ${AUC}_{0\to t,tissue}/{AUC}_{0\to t,blood}$ ) was calculated based on the obtained AUC values. This ratio allowed for the comparison of differential tissue distribution of ammoxetine in rats.

#### Excretion study in rats

Five SD rats were pre-adapted in separate metabolic cages with access to water and a solid diet. They were then administered a single oral dose of 20 mg/kg of ammoxetine after a 12-h fasting period and free access to water. Feces and urine samples were collected at specific time intervals: 0–5 h, 5–12 h, 12–24 h, 24–48 h, and 48–72 h post-administration. The dried fecal weights and urine volumes were measured and recorded at each sampling period. The collected feces were ground, thoroughly mixed, and then homogenized in distilled water to create a 10% homogenate, which was used as the fecal sample. In parallel, after a 12-h fasting period with free access to water, five SD rats were anesthetized, and bile duct cannulation was performed to collect bile samples. Once recovered from anesthesia, these rats received a single oral dose of 20 mg/kg of ammoxetine *via* oral gavage. Bile was collected at the following time points: 0–5 h, 5–12 h, 12–24 h, 24–48 h, and 48–72 h post-dosing, and its volume was measured. All collected samples were stored at −30°C until further processing. For sample processing, 100 μL of feces homogenates, urine, or bile samples were taken and mixed with 100 μL of L-phencynonate (IS, 50 ng/mL) and 300 μL of methanol. After vortexing, the mixture was centrifuged at 13,800×*g* for 10 min. The resulting supernatants were analyzed using an LC-MS-MS method.

### Bioanalytical methods

A comprehensive description of the analytical instruments and conditions used for both *in vivo* and *in vitro* studies can be found in Supplementary Data Sheet 1. The method validation process encompassed various aspects, including specificity, linearity, accuracy and precision (intra-assay and inter-assay), extraction recovery, matrix effect, and stability. These validation parameters were carefully evaluated to ensure the reliability and robustness of the analytical methods employed in this study.

## Results

### Evaluation of BBB permeability through MDCK-MDR1 cell model and molecular docking

**FIGURE 2:**
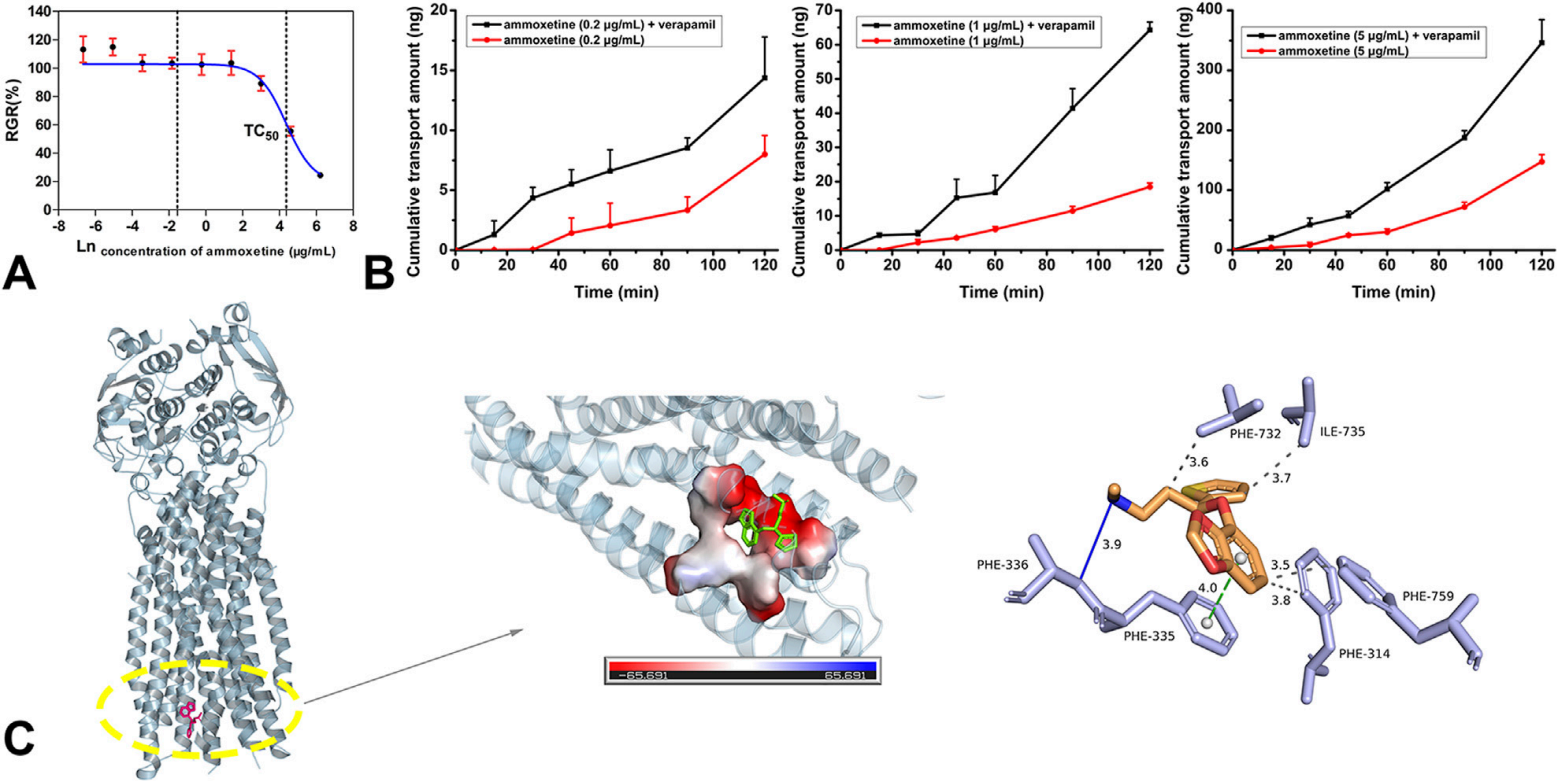
**(A)** The natural logarithm of the ammoxetine concentration-RGR curve (n = 6). **(B)** The effects of verapamil on the apical to basolateral transport of ammoxetine in the MDCK-MDR1 cell line. **(C)** Docking results of ammoxetine and P-gp: docking pocket and three-dimensional interactions.

The MTT assay demonstrated that ammoxetine exhibited a cellular inhibition rate of less than 10% on MDCK-MDR1 cells across the concentration range of 0–20 μg/mL (Supplementary Table S1). The natural logarithm of the ammoxetine concentration-RGR curve yielded a calculated TC_50_ value of (79.76 ± 18.94) μg/mL (Figure 2A). Consequently, for the transmembrane transport experiment of ammoxetine in MDCK-MDR1 cells, working solution concentrations of 0.2, 1, and 5 μg/mL were employed. The transport rate and amount of ammoxetine were found to be positively correlated with the initial concentrations on the loading side, suggesting that the transmembrane transport mechanism of ammoxetine may involve simple diffusion (Figure 2B). The $P_{app\left(A\to B\right)}$ values of ammoxetine at different concentrations on the MDCK-MDR1 cell line ranged from 6.605 × 10^−6^ cm/s to 15.827 × 10^−6^ cm/s (Supplementary Table S2), significantly surpassing the threshold (3 × 10^−6^ cm/s) for facile passage across the BBB (Mander et al., 2023). This indicates a strong ability of ammoxetine to penetrate the brain. The $E_{R}$ values of ammoxetine at different concentrations on MDCK-MDR1 cells ranged from 1.673 to 1.901 (Supplementary Table S2). Furthermore, in the presence of the P-gp inhibitor verapamil, both the transport rate/amount and the $P_{app}$ values of ammoxetine at each concentration from A to B on the MDCK-MDR1 cell line significantly increased (Figure 2B; Supplementary Table S2). These findings collectively suggest that ammoxetine is likely a substrate of P-gp, and P-gp plays a significant role in the efflux of ammoxetine. Molecular docking was performed to further ascertain the binding affinity of ammoxetine with P-gp. The strong binding affinity was indicated by the low energy level (−6.9 kcal/mol). As depicted in Figure 2C, ammoxetine can bind to a deep cavity on the surface of the P-gp protein, exhibiting excellent shape complementarity and a superior binding pattern. Amino acid residues, including Phe 314, Phe 759, Phe 732, and Ile 735, were involved in forming hydrophobic interactions with ammoxetine. Additionally, Phe 335 engaged in π–π stacking interaction with the benzene ring of ammoxetine. Notably, Phe 336 formed hydrogen bonds with the nitrogen atom of ammoxetine, contributing to their close interaction. These findings indicate a strong binding interaction between ammoxetine and P-gp.

### Metabolic profile

The metabolic stability of ammoxetine was assessed in liver microsomes, revealing linear elimination within the range of 0–120 min in both HLM and DLM. However, in RLM and MLM, ammoxetine exhibited linear elimination within the range of 0–15 min (Supplementary Figure S1A). The values of $t_{1/2}$ and *CL*_*int*_ were determined based on the *in vitro* metabolic kinetics study presented in Supplementary Figure S1B and Table 1. It is evident from these results that the metabolism rate of ammoxetine in liver microsomes varies across different species, with MLM > RLM > DLM > HLM. The highest values of *CL*_*int*_ and *CL*_*h*_ were observed in MLM and RLM, respectively. Consequently, the beagle dog demonstrated a closer similarity to humans in terms of clearance rates.

**TABLE 1:** *In vitro* metabolic parameters and predicted hepatic clearance of ammoxetine in liver microsomes of four species (n = 3).

Liver microsomes	Parameters		
	*t*_1/2_ (min)	*CL*_*int*_ (mL·min^-1^·kg^-1^)	*CL*_*h*_ (mL·min^-1^·kg^-1^)
HLM	249.30 ± 6.09	16.08 ± 0.40	9.05 ± 0.13
DLM	138.42 ± 2.96	36.07 ± 0.78	16.64 ± 0.17
MLM	7.57 ± 0.12	618.10 ± 9.67	40.73 ± 0.04
RLM	12.81 ± 0.99	489.11 ± 38.21	49.58 ± 0.39

**FIGURE 3:**
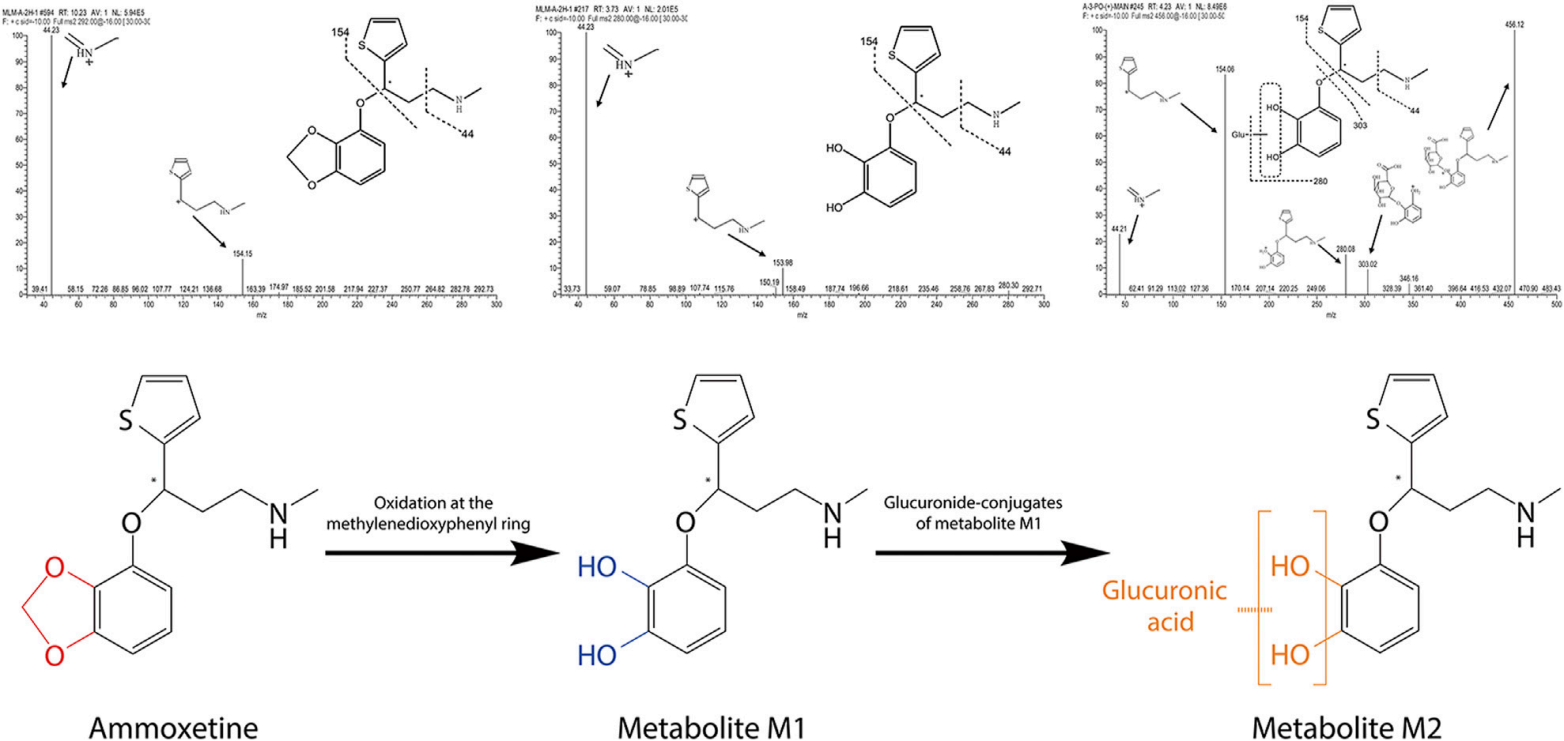
Fragmentation patterns of ammoxetine and identified metabolites obtained through the analysis of their MS/MS fragmentations, along with the proposed main metabolic pathway for ammoxetine.

Two metabolites, M1 and M2, were identified from the MLM incubation samples and rat urine samples, respectively. As shown in Figure 3, in the MLM incubation samples, ammoxetine was detected as its protonated molecule [M + H]^+^ at *m/z* 292 under the Q1 MS full scan mode, with a retention time (RT) of 10.23 min. Through the analysis of its MS/MS fragmentation, two major fragment ions at *m/z* 154 and 44 were observed. The protonated molecules at *m/z* 280 (i.e., [M + H−12]^+^) of metabolite M1, exhibiting the same fragmentation pattern as ammoxetine, corresponded to the oxidation of the methylenedioxyphenyl ring of the parent ion at *m/z* 292. In contrast to the *in vitro* samples, the primary product of ammoxetine in urine samples appeared as a protonated molecule at *m/z* 456 (i.e., [M + H−12 + 176]^+^) under the Q1 MS full scan mode, with an RT of 4.23 min. It generated a series of fragment ions at *m/z* 280, 154, 44, and 303, which were detected under the MS/MS full scan mode. The characteristic fragment ion at *m/z* 280 was likely formed by the loss of 176 Da (glucuronic acid) from the protonated parent ion at *m/z* 456. The neutral loss scan of 176 Da revealed precursor ions at *m/z* 456 and 454 under positive and negative modes, respectively, confirming the presence of a glucuronide. Therefore, metabolite M2 was inferred to be the glucuronide conjugate of metabolite M1, which may undergo rapid conversion to M2 *in vivo*.

**FIGURE 4:**
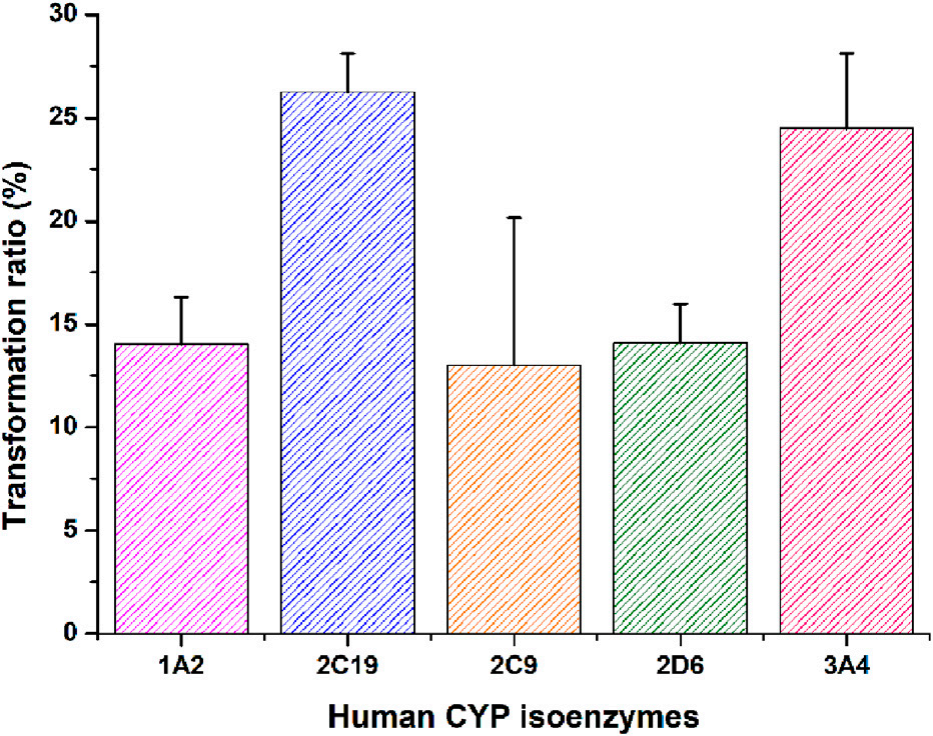
Transformation ratio of ammoxetine in recombinant CYP isoforms (n = 5).

*In vitro* phenotyping studies were conducted to investigate the principal enzymes involved in ammoxetine metabolism. Ammoxetine did not undergo metabolic conversion in the corresponding control groups of the NADPH-free incubation system. In comparison to the zero-time control group, the residual amounts of the parent drug decreased in the various recombinant human CYP enzyme reaction groups. Significant alterations were observed in the residual amounts of the parent drug in the CYP 2C19 and CYP 3A4 enzyme reaction groups compared to the zero-time control group (*p* < 0.05). Among the tested enzymes, CYP 2C19 exhibited the highest metabolic capacity, metabolizing 26.27% of ammoxetine, followed by CYP 3A4, which metabolized 24.49% of the drug. These findings suggest that ammoxetine is primarily metabolized by CYP2C19 and CYP3A4 enzymes in humans (Supplementary Table S3; Figure 4).

### CYP inhibition profile

**FIGURE 5:**
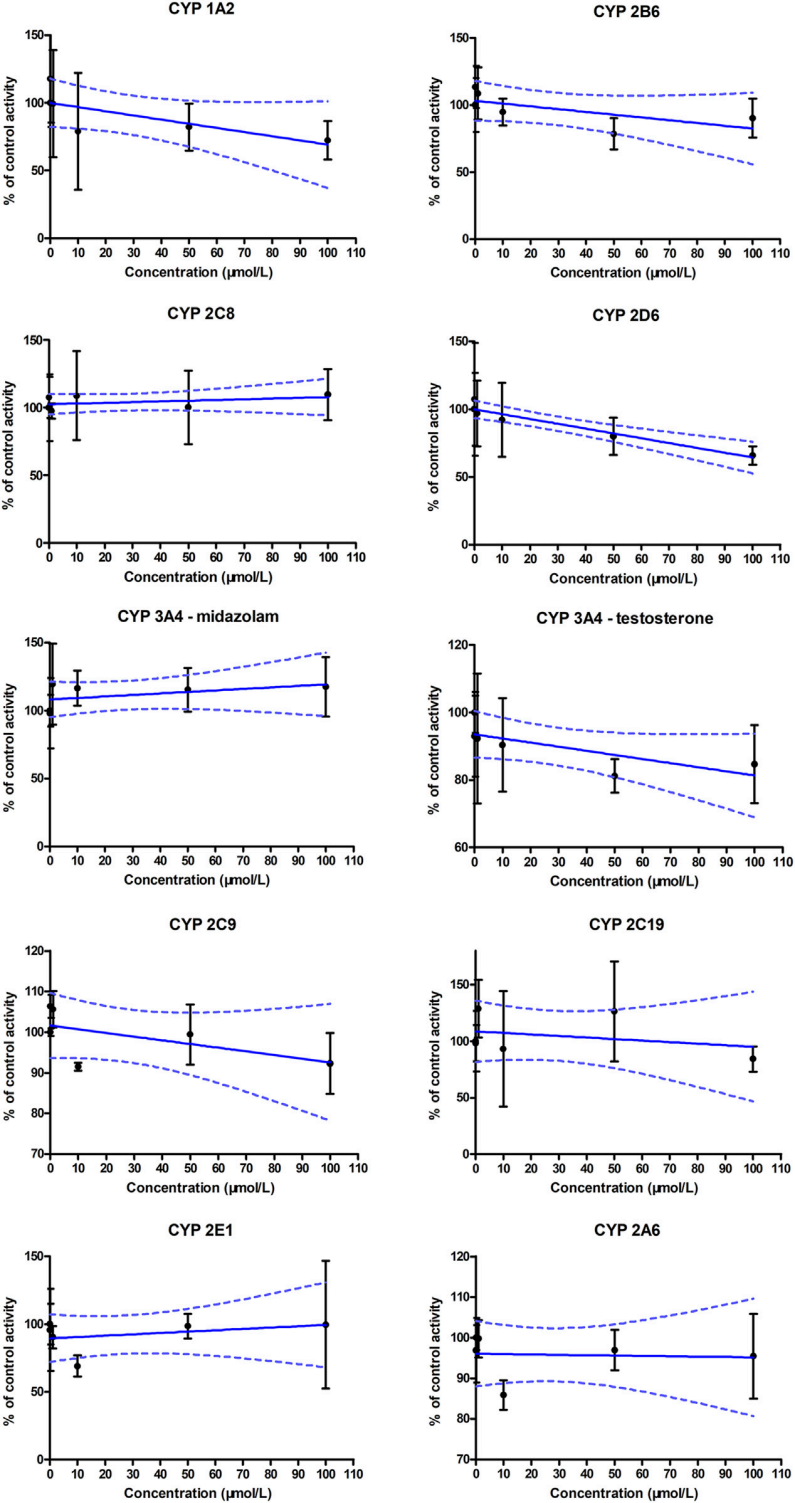
IC_50_ curves for human CYP enzyme activities using the cocktail substrates (n = 3).

The generation of metabolites of probe substrates for CYP enzymes in the blank control group was set as 100%. In the experimental groups, different concentrations of ammoxetine were added to the human liver microsomal incubation system, and the generation of metabolites of probe substrates was compared to the control group. The IC_50_ curves for human CYP enzyme activities are presented in Figure 5. The results indicated no statistically significant differences (*p* > 0.05) in the generation of metabolites of probe substrates for CYP 1A2, CYP 2C9, CYP 2C19, CYP 3A4, CYP 2D6, CYP 2E1, CYP 2C8, CYP 2B6, and CYP 2A6 when exposed to ammoxetine concentrations ranging from 0.1 to 100 μmol/L compared to the blank control. The IC_50_ values of ammoxetine were all greater than 100 μmol/L. Therefore, these findings suggest that ammoxetine exhibits minimal inhibitory effects on these nine CYP enzymes, indicating a low likelihood of ammoxetine exerting inhibitory effects on these CYP enzymes in clinical practice (Mao et al., 2024).

### PPB profile

The PPB rate of ammoxetine was determined to be approximately 50%–60% in beagle dogs, rats, and humans, indicating a moderate level of binding (Table 2). There were no obvious differences observed in the concentration factor or the species factor between groups. These findings suggest that the PPB of ammoxetine remains consistent across different species and is not influenced by concentration.

**TABLE 2:** Percentage of ammoxetine-protein binding rate in beagle dogs, rats, and humans (n = 3).

Concentration of ammoxetine (ng/mL)	Percentage of ammoxetine-protein binding rate (%)		
	Beagle dogs	Rats	Humans
50	53.62 ± 1.93	66.16 ± 1.69	58.51 ± 2.54
150	53.21 ± 4.96	55.35 ± 6.30	49.71 ± 1.91
450	57.74 ± 5.20	49.42 ± 9.65	55.01 ± 4.49

### Configurational stability

A chiral chromatographic column was employed to separate the enantiomers, and the RT of X-071031B (racemic 071031B) and S-071031B (ammoxetine) standards were determined for identification. Plasma samples were collected from rats 2 h after oral administration of 50 mg/kg of S-071031B or R-071031B. The presence of a chromatographic peak at the RT corresponding to the opposite enantiomer following the administration of the S-isomer would indicate the conversion of the S-isomer to the R-isomer in rats. Conversely, the absence of such conversion would suggest that the S-isomer does not undergo conversion to the R-isomer in rats. The same principle applies to investigating the conversion of the R-isomer to the S-isomer.

**FIGURE 6:**
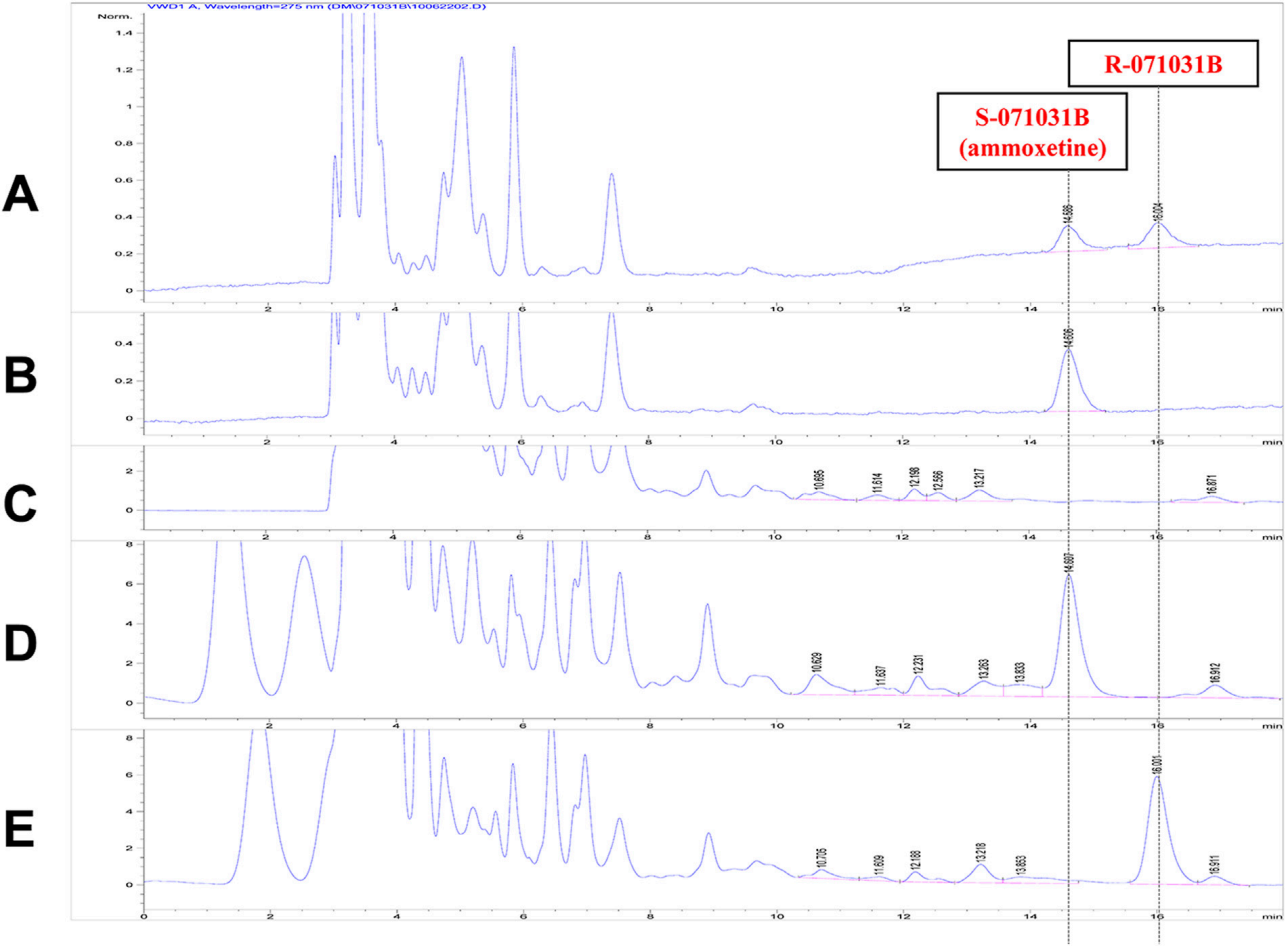
HPLC chromatograms: **(A)** Standard solution of X-071031B (5 μg/mL) **(B)** Standard solution of S-071031B (i.e., ammoxetine); (5 μg/mL); **(C)** Blank plasma from rats; **(D)** Plasma sample from rats 2 h after oral administration of S-071031B (50 mg/kg); **(E)** Plasma sample from rats 2 h after oral administration of R-071031B (50 mg/kg).

The study findings revealed that only a chromatographic peak with an RT of 14.6 min was observed in the plasma samples of rats after the oral administration of S-071031B for 2 h. No chromatographic peak corresponding to R-071031B was detected (Figure 6). This indicates that S-071031B does not undergo conversion to R-071031B in rats. Similarly, after the oral administration of R-071031B for 2 h, only a chromatographic peak with an RT of 16.0 min was observed in the plasma samples of rats, and no chromatographic peak corresponding to S-071031B was detected (Figure 6). This demonstrates that R-071031B does not convert to S-071031B in rats.

### Method development and validation for *in vivo* studies

LC-MS-MS methods were developed and validated to accurately quantify ammoxetine in rat and beagle dog plasma, as well as in rat tissues, urine, feces, and bile. The methods involved direct protein precipitation using methanol, providing a straightforward and rapid sample preparation process with high extraction recovery rates (absolute recoveries >85% in all matrices). Importantly, no endogenous interferences were observed in blank biological samples from rats and beagle dogs, ensuring precise determination of both ammoxetine and the IS. The linearity of the methods was exceptional, demonstrating a robust linear relationship between the concentration of the analyte and the corresponding response. The precision and accuracy of the plasma methods were evaluated through intra-day and inter-day analyses, yielding satisfactory results. Additionally, stability tests conducted in rat and beagle dog plasma met the analytical requirements for determination. Furthermore, the majority of the analytes met the necessary criteria for matrix effects when quantifying ammoxetine in biological samples. The developed methods are well-suited for determining ammoxetine concentrations in rat and beagle dog biological samples, enabling *in vivo* studies of this compound. They provide specific and highly sensitive quantification, facilitating investigations into the drug’s behavior *in vivo*. Detailed results regarding the establishment and validation of these analytical methods can be found in Supplementary Data Sheet 2.

### PK profile

**FIGURE 7:**
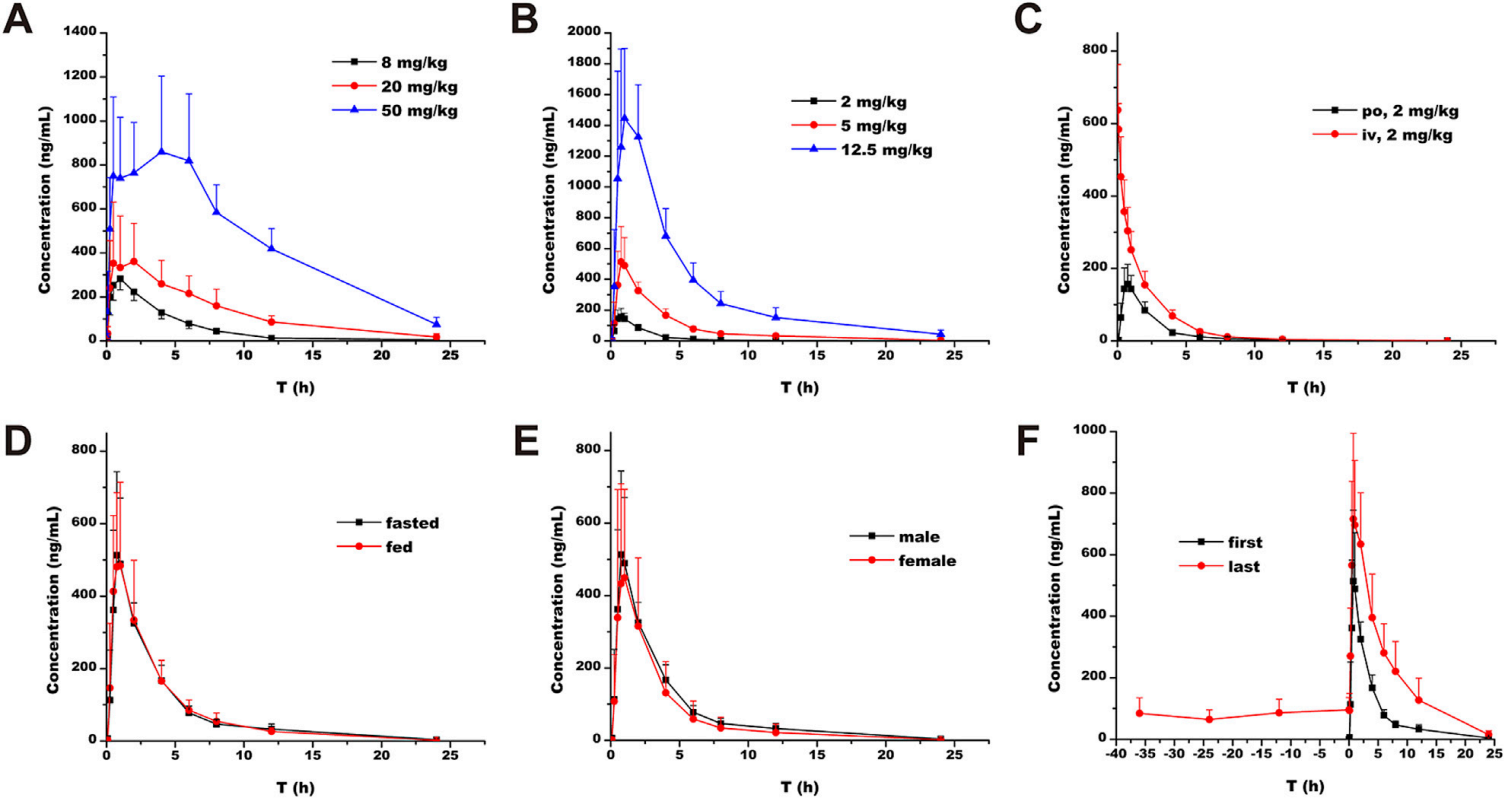
Plasma concentration-time profiles of ammoxetine: **(A)** Rats (n = 6) - Oral administration at doses of 8, 20, and 50 mg/kg; **(B)** Beagle dogs (n = 5) - Oral administration at doses of 2, 5, and 12.5 mg/kg; **(C)** Beagle dogs (n = 5) - Oral administration and intravenous injection at a dose of 2 mg/kg; **(D)** Beagle dogs (n = 5) - Oral administration at a dose of 5 mg/kg in fasted and fed conditions; **(E)** Beagle dogs (n = 5) - Oral administration at a dose of 5 mg/kg in male and female dogs; **(F)** Beagle dogs (n = 5) - Multiple oral administrations at a dose of 5 mg/kg.

Single-dose oral administration of ammoxetine at low, medium, and high doses was performed on rats and beagle dogs. The results demonstrated rapid absorption, with detectable blood drug concentrations as early as 5 min after administration (Figure 7A; Figure 7B). The T_max_ values ranged from 0.75 to 3.83 h in rats and 0.75–1.40 h in beagle dogs (Table 3). Statistical analysis revealed significant differences (*p* < 0.05) in the pharmacokinetic parameters MRT_(0-t)_, MRT_(0-∞)_, and t_1/2/z_ among the different groups for rats, and MRT_(0-t)_, MRT_(0-∞)_, and CL_z_/F among the different groups for beagle dogs. Moreover, the AUC and C_max_ increased with higher doses. The ratios of AUC_(0-t)_ and C_max_ for the three oral doses were 1: 2.34: 8.06 and 1: 1.39: 3.44, respectively, for rats, and 1: 4.68: 18.91 and 1: 3.17: 8.91, respectively, for beagle dogs. Additionally, in rats, as the dose increased, MRT_(0-∞)_, MRT_(0-t)_, and t_1/2/z_ gradually increased, suggesting a potential saturation of drug elimination. In beagle dogs, MRT_(0-∞)_ and MRT_(0-t)_ also increased with higher doses, while CL_z_/F decreased, indicating a potential saturation of drug elimination as well (Table 3).

**TABLE 3:** Pharmacokinetic parameters of ammoxetine in rats following oral administration at doses of 8, 20, and 50 mg/kg (mean ± SD, n = 6) and in beagle dogs following oral administration at doses of 2, 5, and 12.5 mg/kg (mean ± SD, n = 5).

Parameter	Rats				Beagle dogs			
	8 mg/kg	20 mg/kg	50 mg/kg	*p*-value	2 mg/kg	5 mg/kg	12.5 mg/kg	*p*-value
AUC_(0-t)_/(μg/L·h)	1,366.14 ± 251.62	3,200.20 ± 1,308.69	11,011.21 ± 2,868.52	-	417.73 ± 107.78	1955.33 ± 526.60	7,899.46 ± 2022.29	-
AUC_(0-∞)_/(μg/L·h)	1,372.58 ± 255.30	3,402.71 ± 1,244.95	12,948.54 ± 4,710.24	-	419.81 ± 108.04	1978.88 ± 532.81	8,395.25 ± 1817.57	-
MRT_(0-t)_/h	4.38 ± 0.47	6.73 ± 1.25	7.74 ± 0.52	0.00001	2.94 ± 0.55	4.40 ± 0.65	5.43 ± 1.18	0.00179
MRT_(0-∞)_/h	4.49 ± 0.44	8.57 ± 3.58	11.84 ± 7.34	0.00189	3.08 ± 0.69	4.70 ± 0.85	7.53 ± 4.70	0.00701
t_1/2z_/h	2.87 ± 0.59	5.61 ± 2.59	7.51 ± 5.70	0.00749	3.40 ± 1.28	3.81 ± 0.52	6.20 ± 4.43	0.32956
T_max_/h	0.75 ± 0.27	1.58 ± 1.39	3.83 ± 2.70	0.19522	0.75 ± 0.18	0.80 ± 0.11	1.40 ± 0.60	0.05918
CL_z_/F(L/h/kg)	6.01 ± 1.2	6.49 ± 2.10	4.34 ± 1.65	0.10104	5.01 ± 1.22	2.71 ± 0.86	1.55 ± 0.37	0.00397
V_z_/F(L/kg)	24.44 ± 4.48	53.53 ± 28.97	40.28 ± 16.45	0.06141	24.95 ± 12.99	14.87 ± 5.08	13.90 ± 11.03	0.10228
C_max_/(μg/L)	287.19 ± 50.77	399.75 ± 246.16	987.12 ± 361.76	-	165.18 ± 49.55	523.70 ± 214.46	1,472.40 ± 446.49	-

Note: AUC_(0-t)_ represents the area under the plasma concentration-time curve from time zero to the last observed time point. AUC_(0-∞)_ represents the area under the plasma concentration-time curve from time zero to infinity. MRT_(0-t)_ represents the mean residence time from time zero to the last observed time point. MRT_(0-∞)_ represents the mean residence time from time zero to infinity. t_1/2z_ represents the terminal elimination half-life. C_max_ represents the maximum plasma concentration. T_max_ represents the time to reach C_max_. CL_z_/F represents the apparent clearance. V_z_/F represents the apparent volume of distribution.

The calculated *F* of ammoxetine, based on AUC_(0-t)_ and AUC_(0-∞)_, was (41.76 ± 9.25)% and (41.94 ± 9.31)%, respectively (Table 4). In particular, the estimated total clearance and volume of distribution were (2.04 ± 0.36) L/h/kg and (7.82 ± 1.20) L/kg, respectively (Table 4). These results indicate a relatively high oral bioavailability for ammoxetine (Figure 7C; Table 4). When comparing the administration of the drug under fasting and non-fasting conditions, statistically significant differences were observed only in the t_1/2z_ parameter (*p* < 0.05), while the other parameters did not exhibit statistically significant differences (*p* > 0.05) (Figure 7D; Table 4). These findings suggest that diet has no significant impact on the pharmacokinetic processes of ammoxetine in beagle dogs. Similarly, no statistically significant differences (*p* > 0.05) were observed in the major pharmacokinetic parameters between male and female beagle dogs, indicating no gender differences in the pharmacokinetic profile of ammoxetine in beagle dogs (Figure 7E; Table 4).

**TABLE 4:** Pharmacokinetic parameters of ammoxetine in beagle dogs from three separate studies following oral administration at doses of 2 mg/kg, 5 mg/kg, and 5 mg/kg, respectively (mean ± SD, n = 5).

Parameter	Study on absolute bioavailability (dose: 2 mg/kg)			Evaluation of the impact of nonfasting on PK (dose: 5 mg/kg)			Comparison of sex differences in PK (dose: 5 mg/kg)		
	Oral administration	Intravenous administration	*p*-value	Fasted	Fed	*p*-value	Male	Female	*p*-value
AUC_(0-t)_/(μg/L·h)	417.73 ± 107.78	1,007.57 ± 183.79	0.00160	1955.33 ± 526.60	1947.89 ± 738.46	0.95442	1955.33 ± 526.60	1,643.85 ± 1,143.70	0.56930
AUC_(0-∞)_/(μg/L·h)	419.81 ± 108.04	1,008.53 ± 184.44	0.00167	1978.88 ± 532.81	1958.31 ± 741.90	0.87499	1978.88 ± 532.81	1,656.43 ± 1,160.77	0.56561
MRT_(0-t)_/h	2.94 ± 0.55	2.30 ± 0.29	0.01585	4.40 ± 0.64	4.14 ± 0.54	0.1624	4.40 ± 0.64	3.74 ± 0.78	0.19631
MRT_(0-∞)_/h	3.08 ± 0.69	2.33 ± 0.29	0.0317	4.70 ± 0.85	4.28 ± 0.59	0.09746	4.70 ± 0.85	3.88 ± 0.83	0.18639
t_1/2z_/h	3.40 ± 1.28	2.72 ± 0.51	0.43413	3.81 ± 0.52	3.28 ± 0.28	0.04456	3.81 ± 0.52	3.45 ± 0.40	0.39013
T_max_/h	0.75 ± 0.18	0.05 ± 0.03	0.00096	0.80 ± 0.11	0.80 ± 0.21	0.96486	0.80 ± 0.11	0.85 ± 0.22	0.48842
CL_z_/F(L/h/kg)	5.01 ± 1.22	-	0.00323	2.71 ± 0.86	2.96 ± 1.37	0.35669	2.71 ± 0.86	4.29 ± 2.66	0.28241
V_z_/F(L/kg)	24.95 ± 12.99	-	0.04665	14.87 ± 5.08	13.97 ± 6.80	0.4803	14.87 ± 5.08	20.87 ± 12.27	0.35832
CL_z_ (L/h/kg)	-	2.04 ± 0.36	-	-	-	-	-	-	-
V_z_ (L/kg)	-	7.82 ± 1.20	-	-	-	-	-	-	-
C_max_/(μg/L)	165.18 ± 49.55	649.01 ± 118.97	0.00114	523.70 ± 214.46	521.54 ± 234.23	0.88517	523.70 ± 214.46	475.78 ± 284.08	0.64492

Note: AUC_(0-t)_ represents the area under the plasma concentration-time curve from time zero to the last observed time point. AUC_(0-∞)_ represents the area under the plasma concentration-time curve from time zero to infinity. MRT_(0-t)_ represents the mean residence time from time zero to the last observed time point. MRT_(0-∞)_ represents the mean residence time from time zero to infinity. t_1/2z_ represents the terminal elimination half-life. C_max_ represents the maximum plasma concentration. T_max_ represents the time to reach C_max_. CL_z_/F represents the apparent clearance. V_z_/F represents the apparent volume of distribution.

In the context of multiple oral administrations, the trough concentrations of ammoxetine before the 4th, 5th, 6th, and 7th doses were (84.17 ± 50.58), (64.21 ± 31.71), (86.13 ± 43.95), and (95.71 ± 39.83) ng/mL, respectively (Figure 7F; Table 5). Statistical analysis revealed that these differences were not statistically significant (*p* = 0.69146), indicating the attainment of steady-state concentrations after the 4th oral administration. No statistically significant differences (*p* > 0.05) were observed in the pharmacokinetic parameters t_1/2z_ and T_max_ between the first and last doses. However, other parameters such as C_max_, V_z_/F, CL_z_/F, MRT_(0-∞)_, AUC_(0-∞)_, MRT_(0-t)_, and AUC_(0-t)_ exhibited statistically significant differences (*p* < 0.05) (Figure 7F; Table 5). These findings suggest a potential trend of drug accumulation in beagle dogs following multiple oral administrations of ammoxetine.

**TABLE 5:** Pharmacokinetic parameters of ammoxetine in beagle dogs following multiple oral administrations with a dose of 5 mg/kg (mean ± SD, n = 5).

Parameter	First time	Last time	*p*-value
AUC_SS_/(μg/L·h)	-	4,046.33 ± 1,347.23	-
AUC_(0-t)_/(μg/L·h)	1955.33 ± 526.60	4,900.92 ± 1813.18	0.00804
AUC_(0-∞)_/(μg/L·h)	1978.88 ± 532.81	5,000.83 ± 1890.01	0.00895
MRT_(0-t)_/h	4.40 ± 0.64	5.76 ± 0.60	0.00398
MRT_(0-∞)_/h	4.70 ± 0.85	6.21 ± 0.73	0.01718
t_1/2z_/h	3.81 ± 0.52	4.13 ± 0.85	0.60597
T_max_/h	0.80 ± 0.11	1.10 ± 0.52	0.28351
CL_z_/F(L/h/kg)	2.71 ± 0.86	1.12 ± 0.41	0.00168
V_z_/F(L/kg)	14.87 ± 5.08	6.63 ± 2.77	0.00768
C_max_/(μg/L)	523.70 ± 214.46	760.62 ± 253.99	0.01282
C_min_/(μg/L)	-	95.71 ± 39.83	-
C_av_/(μg/L)	-	337.19 ± 112.27	-
DF	-	2.00 ± 0.45	-

Note: AUC_SS_, represents the area under the plasma concentration-time curve at steady-state during a dosing interval. AUC_(0-t)_ represents the area under the plasma concentration-time curve over the specified sampling period. AUC_(0-∞)_ represents the area under the plasma concentration-time curve from time zero to infinity. MRT_(0-t)_ represents the mean residence time over the specified sampling period. MRT_(0-∞)_ represents the mean residence time from time zero to infinity. t_1/2z_ represents the terminal elimination half-life. C_max_ represents the maximum plasma concentration. C_min_ represents the minimum plasma concentration. T_max_ represents the time to reach C_max_. CL_z_/F represents the apparent clearance. V_z_/F represents the apparent volume of distribution. C_av_ represents the average plasma concentration within a dosing interval. DF, represents the degree of fluctuation.

### Tissue distribution profile

**FIGURE 8:**
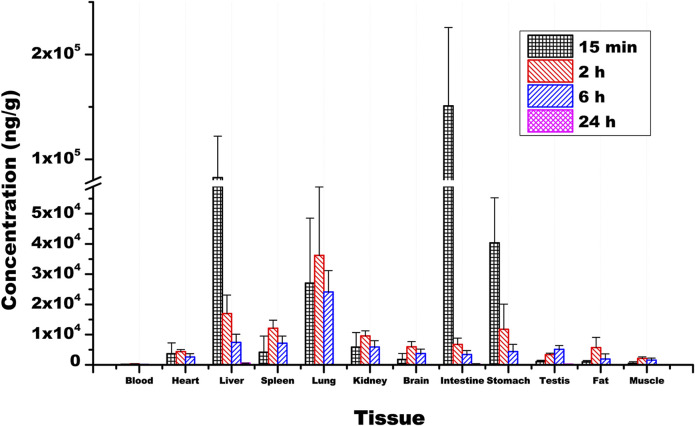
Mean concentrations of ammoxetine in rat tissues (n = 6) following oral administration at a dose of 20 mg/kg. Note: Plasma concentration is measured in ng/mL.

Ammoxetine exhibits wide distribution in various tissues within the rat’s body. At 15 min post-administration, the highest concentrations were observed in the intestine, liver, and stomach samples, possibly due to the oral administration route. Except for the testis, where the drug reached its peak at 6 h, peak concentrations in other tissues including the heart, spleen, lung, kidney, brain, fat, and muscle were reached at 2 h (Figure 8). The concentrations of the drug were relatively high in the liver, lung, kidney, and spleen, which can be attributed to the rich blood supply in these tissues. Two hours after administration, the order of drug concentrations in tissues, from highest to lowest, was as follows: lung, liver, spleen, stomach, kidney, intestine, brain, fat, heart, testis, muscle, and blood. The significant distribution of ammoxetine in brain tissue suggests its ability to cross the BBB. After 24 h of administration, drug concentrations in all tissues decreased to low levels, indicating no tissue accumulation effect (Figure 8). The tissue-to-blood AUC ratio for each tissue is depicted in Table 6, presenting the tissue exposure in descending order: lung > liver > intestine > stomach > spleen > kidney > testis > brain > heart > fat > muscle > blood. Plasma exposure only accounted for approximately 0.186% of the total systemic exposure (calculated as ${AUC}_{0\to t,blood}/{AUC}_{0\to t,total}$ ).

**TABLE 6:** Comparison of AUC_(0→t)_ of ammoxetine in rat tissues (mean ± SD, n = 6).

Tissue	AUC_(0→t)_/(ng/g·h)	AUC_(0→t),tissue_/AUC_(0→t),blood_
Blood	2,615.20 ± 408.26	-
Heart	44,743.11 ± 11,086.06	17.11 ± 3.30
Liver	208,081.20 ± 64,872.68	81.37 ± 26.76
Spleen	117,673.30 ± 31,479.36	46.03 ± 14.18
Lung	393,670.80 ± 31,407.40	154.85 ± 34.69
Kidney	98,000.01 ± 25,400.16	37.66 ± 8.18
Brain	60,541.26 ± 19,438.07	23.26 ± 6.19
Intestine	190,710.5 ± 52,982.23	75.61 ± 28.20
Stomach	118,279.40 ± 37,565.21	48.76 ± 27.59
Fat	38,592.32 ± 24,498.69	14.15 ± 7.84
Muscle	24,154.50 ± 6,583.59	9.31 ± 2.29
Testis	69,347.84 ± 13,459.91	27.32 ± 8.12

Note: The unit for AUC_(0→t)_ in blood is μg/L·h.

### Excretion profile

**FIGURE 9:**
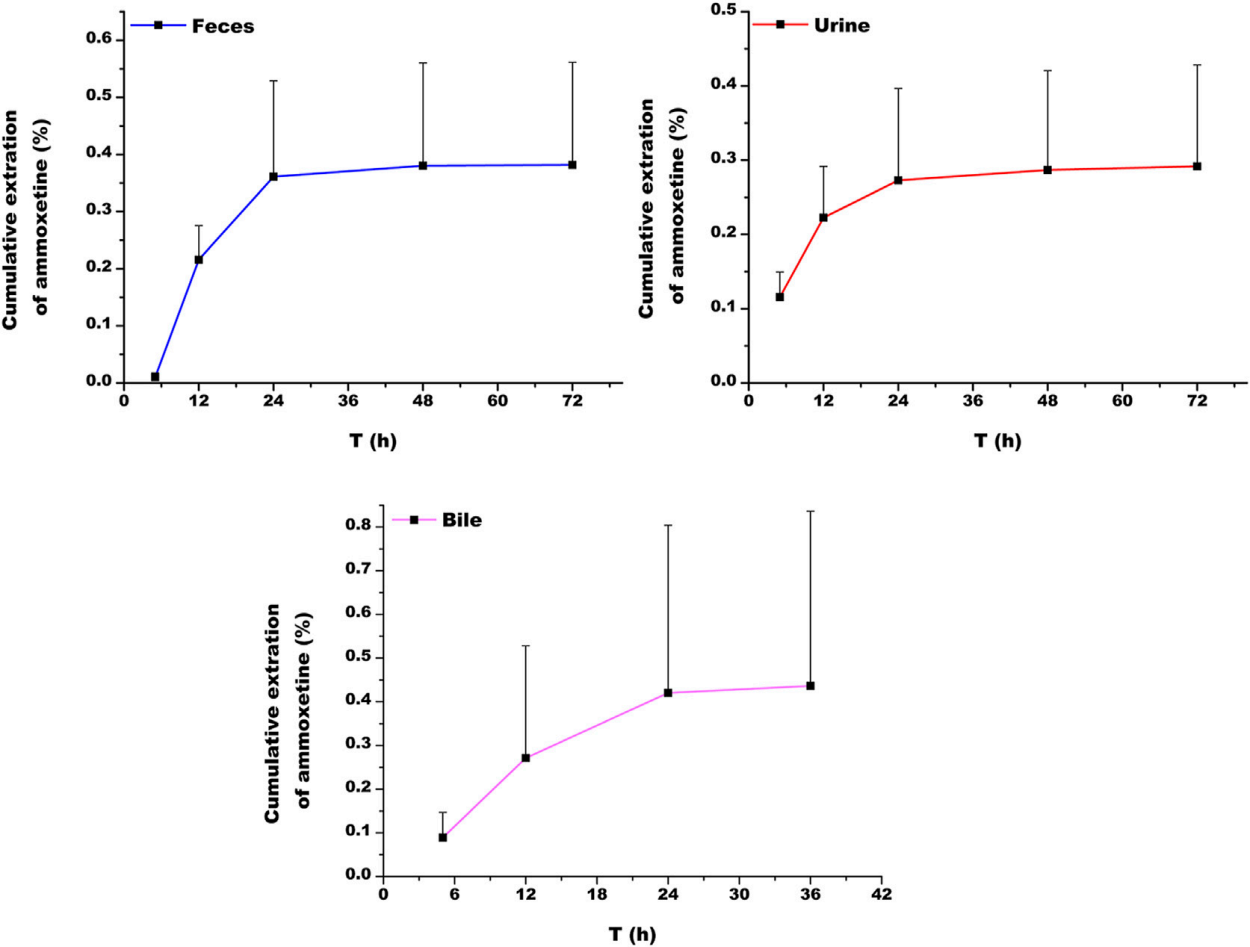
Cumulative excretion of ammoxetine in feces, urine, and bile in rats (n = 5) after oral administration at a dose of 20 mg/kg.

At 72 h following oral administration of ammoxetine (20 mg/kg), the cumulative excretion rates of the drug prototype in feces, urine, and bile samples of rats accounted for (0.382 ± 0.180)%, (0.292 ± 0.137)%, and (0.436 ± 0.400)%, respectively, of the total drug administration (Figure 9; Table 7). The total cumulative excretion rate accounted for approximately 1.11%, indicating that ammoxetine is likely primarily converted into metabolites after administration and subsequently excreted. The main elimination pathway *in vivo* appears to be excretion after metabolic transformation.

**TABLE 7:** Excretion (%) of ammoxetine in feces, urine, and bile after oral administration with a dose of 20 mg/kg in rats (mean ± SD, n = 5).

Time (h)	Feces		Urine		Bile	
	Excretion rate	Cumulative excretion rate	Excretion rate	Cumulative excretion rate	Excretion rate	Cumulative excretion rate
0–5	0.010 ± 0.008	0.010 ± 0.008	0.116 ± 0.034	0.116 ± 0.034	0.089 ± 0.058	0.089 ± 0.058
5–12	0.206 ± 0.056	0.216 ± 0.060	0.107 ± 0.048	0.223 ± 0.069	0.182 ± 0.212	0.271 ± 0.257
12–24	0.145 ± 0.126	0.361 ± 0.168	0.050 ± 0.057	0.273 ± 0.124	0.149 ± 0.146	0.420 ± 0.384
24–48	0.019 ± 0.016	0.380 ± 0.180	0.014 ± 0.010	0.287 ± 0.134	0.149 ± 0.146	0.420 ± 0.384
48–72	0.002 ± 0.003	0.382 ± 0.180	0.005 ± 0.003	0.292 ± 0.137	0.016 ± 0.016	0.436 ± 0.400

## Discussion

Previous studies have provided evidence supporting the strong antidepressant effects and low toxicity of ammoxetine, a novel SNRI, both *in vivo* and *in vitro* (Liu et al., 2022; Xue et al., 2013a; Zhang et al., 2016; Zhang et al., 2018). This paper presents a comprehensive nonclinical pharmacokinetic evaluation of ammoxetine, conducted prior to its entry into clinical trials. This evaluation aimed to obtain *in vivo* and *in vitro* pharmacokinetic data, as well as assess its metabolic properties. It is worth noting that Phase I clinical trials, which have already been published, demonstrated the safety and favorable tolerability profile of ammoxetine in healthy Chinese subjects (Shen et al., 2021). Nevertheless, the preclinical DMPK properties of ammoxetine have not been adequately characterized. Our study aims to address this knowledge gap in the existing literature.

Our *in vivo* tissue distribution analysis revealed that the exposure of ammoxetine in rat brain tissue was approximately 23.26 times higher than that in plasma. This indicates that ammoxetine may effectively penetrate the BBB to reach target organs and exert pharmacological effects. Our study also investigated the permeability of ammoxetine across the BBB using the MDCK-MDR1 cell model and molecular docking. In contrast to duloxetine, which is known as a P-gp inhibitor (Ruike et al., 2010), our findings indicated that ammoxetine acts as a substrate with significant permeability across the BBB, showing promise in effectively reaching the brain. A direct relationship between transport rate and initial concentration tended to show that ammoxetine may well cross the BBB through the simplest means of diffusion and this is a process that does not require energy expenditure. Additionally, our study underscored the role of P-gp, a critical efflux transporter in the BBB, in the transport of ammoxetine. The raised transport rate and the $P_{app\left(A\to B\right)}$ values obtained in the presence of verapamil, a P-gp inhibitor, supported the idea of efflux of ammoxetine by P-gp from the brain. Molecular docking results further revealed a strong binding affinity of ammoxetine to P-gp, indicated by its low energy level, suggesting a stable interaction between the drug and the transporter. Through the docking analysis, it is brought to light that ammoxetine settled in a deep pocket of the P-gp protein and interacts with specific amino acid residues and the outcomes extend as far as hydrophobic interactions and π–π stacking leading to high binding affinity. These findings suggested that P-gp efflux may hinder ammoxetine’s brain penetration. This is a barrier that can be addressed, for instance, by co-administration with P-gp inhibitors, which could enhance the impact of ammoxetine in the brain (Ilyas-Feldmann et al., 2024). The study provides valuable insights into ammoxetine’s BBB permeability. Nevertheless, further work is needed to confirm these findings *in vivo* and investigate whether P-gp blockade affects ammoxetine’s PK and clinical effectiveness.

Chiral safety, particularly the *in vivo* inversion of chiral drugs, represents a significant research focus in PK. Chiral inversion is a highly intricate phenomenon that can potentially occur throughout various stages, including absorption, distribution, metabolism, and excretion (Chen et al., 2020; McVicker and O'Boyle, 2024). Duloxetine is utilized in therapy in its pure enantiomeric form, S-duloxetine, exhibiting twice the activity compared to R-duloxetine (Lupu and Hancu, 2021). Similar to duloxetine, ammoxetine has been demonstrated as a more potent enantiomer, displaying increased efficacy in *in vivo* behavioral assessments while maintaining equivalent potency *in vitro* assays when compared to R-071031B (Xue et al., 2017). Previous studies have shown that the ammoxetine enantiomers did not undergo chiral inversion in dogs and displayed pharmacokinetic variances in both rats and dogs (Li et al., 2014; Yu et al., 2017). Our research further corroborated this by demonstrating that the racemization of ammoxetine did not take place in rats.

Our research also underscores the species-specific variations in the metabolism of ammoxetine, with the most rapid clearance rates observed in MLM and RLM. Factors such as genetic variation may play significant roles in metabolic stability. All proteins within the CYP superfamily share conserved positions across species, yet even minor differences in a few amino acids can profoundly impact substrate selectivity and catalytic turnover of the enzyme (Nishimuta et al., 2013). Differences in the isoform distribution, expression, and activity of CYP enzymes in animals and humans have been demonstrated particularly in the 2C and 3A subfamilies of CYP which are composed of species-specific isoforms (Tian et al., 2021). Among these enzymes, CYP2C19 and CYP3A4, the primary enzymes involved in ammoxetine metabolism, display significant interspecies differences in catalytic activity (Tian et al., 2021). This variability underscores the need for cautious extrapolation of preclinical findings to humans. Nevertheless, the beagle dog showed a higher resemblance to humans in terms of metabolism rates. This was strongly supported by our *in vivo* pharmacokinetic investigations, revealing a significant decrease in CL_z_/F at higher doses during single-dose oral administration of ammoxetine in beagle dogs. Additionally, a notable decrease in CL_z_/F was observed in the initial doses compared to the last doses in multiple oral administrations of ammoxetine. These observations are in concordance with a phase I study by Shen et al. (2021) and support the results by Nishimuta et al. (2013), who stated that dogs demonstrated metabolic activity most closely resembling that of humans compared to rats. Except for beagle dogs, MRT_(0-∞)_, MRT_(0-t)_, and t_1/2/z_ all increased gradually with the increase of dosage in rats in pharmacokinetic studies, suggesting that ammoxetine in rats may exhibit a trend of drug saturation elimination with increasing dosage. Overall, this indicates the characteristics of ammoxetine, potentially displaying saturation in drug elimination and a tendency toward drug accumulation. One putative explanation is that CYP2C19 and CYP3A4, the primary human enzymes responsible for ammoxetine metabolism, could become saturated when clearing ammoxetine. This saturation may lead to non-linear PK for ammoxetine following multiple doses, as reflected by reference (Shen et al., 2021). This data is also crucial for predicting potential drug-drug interactions when CYP2C19 and CYP3A4 inhibitors or inducers are co-administered, as well as for understanding the impact of individual genetic variations in CYP enzymes on ammoxetine metabolism. Nonetheless, Shen et al. (2021) failed to establish the influence of *CYP2C19* polymorphisms on ammoxetine metabolism, possibly due to the limited sample size in their study.

Duloxetine undergoes extensive metabolism, leading to the formation of multiple metabolites primarily excreted in conjugated form in the urine, followed by other conjugated forms in plasma and feces (Lantz et al., 2003). Similarly, our study identified two major metabolites of ammoxetine, M1 and M2. M1 is generated through oxidation of the methylenedioxyphenyl ring, likely a primary biotransformation pathway of ammoxetine involving CYP2C19 and CYP3A4, the main human enzymes responsible for liver metabolism. On the other hand, M2, the primary metabolite found in urine, is the glucuronide conjugate of M1. While M2 was detected in both urine and plasma, M1 was not found in plasma (unpublished results), indicating rapid *in vivo* biotransformation of ammoxetine. This highlights glucuronidation as a significant metabolic route for ammoxetine. The cumulative excretion rates in urine, feces, and bile in rats accounted for 1.11% of the total drug administered, indicating the extensive transformation of ammoxetine into metabolites within the body, followed by excretion post-metabolic processing. However, unlike duloxetine, these excretion pathways may not include feces, as reported in the clinical study by Shen et al. (2021), where ammoxetine or its metabolites were not detected in human feces, suggesting enhanced bioavailability of ammoxetine. Indeed, our investigation demonstrated a high absolute bioavailability of ammoxetine (approximately 42%) at a dose of 2 mg/kg in beagle dogs. In a prior preliminary experiment by our team, the absolute bioavailability of ammoxetine was found to be higher than that of duloxetine (approximately 33%) at the same dose in beagle dogs, with minor individual variations (unpublished results). Ammoxetine metabolism is similar to that of paroxetine, a highly specific selective 5-HT reuptake inhibitor (SSRI), likely due to the methylenedioxyphenyl ring structure, which extensively oxidizes into the main inactive metabolites (Bourin et al., 2001). These compounds show no significant inhibitory effects on 5-HT or NE reuptake. Therefore, it is reasonable to infer that neither M1 nor M2 are likely to be pharmacologically active metabolites, as the *in vivo* pharmacological profile remains unaltered by ammoxetine metabolism (Bourin et al., 2001).

Our *in vitro* CYP enzyme inhibition study demonstrated that ammoxetine exhibited minimal inhibitory effects on a range of CYP enzymes, including CYP1A2, CYP2C9, CYP2C19, CYP3A4, CYP2D6, CYP2E1, CYP2C8, CYP2B6, and CYP2A6. This suggests a low likelihood of drug-drug interactions related to CYP inhibition. However, further investigation, including *in vivo* analysis of ammoxetine inhibition or induction of CYP enzymes or transporters, is needed to assess potential interactions with other drugs. Specifically, although the IC_50_ values of ammoxetine for these CYP enzymes were all greater than 100 μmol/L, at 100 μmol/L, the probe substrate levels of CYP2D6 and CYP1A2 decreased to approximately 65.86% and 72.30% of the original blank group, respectively. This implies that ammoxetine may have very weak CYP inhibition on both CYP2D6 and CYP1A2 (Zhao et al., 2019); however, the probability of pharmacokinetic interactions with other drugs based on this inhibition seems to be low. A shared characteristic between paroxetine and ammoxetine is their methylenedioxyphenyl moiety, which can undergo oxidation by CYP2D6, resulting in the formation of a carbene intermediate. This intermediate then irreversibly binds to CYP2D6, leading to its inactivation. This process helps elucidate the experimentally determined inhibition activity of ammoxetine (Don and Smieško, 2020).

The PPB rate of ammoxetine is approximately 50%–60% across beagle dogs, rats, and humans, indicating moderate protein binding. This suggests that a significant portion of the drug is bound to plasma proteins, which could influence its distribution and elimination. The tissue distribution study showed that ammoxetine rapidly and extensively spread to major tissues in rats after gavage administration of 20 mg/kg. The concentration of ammoxetine peaked in most tissues 2 hours post-administration and then decreased to low levels after 24 h, without any tissue accumulation observed. Initially, the highest drug concentrations were found in the intestine and stomach, reflecting the primary absorption site after oral intake, with T_max_ in blood reaching around 2 h. It displayed rapid distribution in organs like the liver, kidney, and spleen, which have strong blood flow, likely tied to *in vivo* blood perfusion (Currie, 2018). This implies that there is a need to be alert when assessing the functional changes of organs with high drug concentrations, such as the lung, liver, spleen, stomach, and kidney, for drug safety assessments. The safety and tolerability assessment of ammoxetine in healthy humans revealed that nausea, pyuria, proteinuria, and vomiting were the most common adverse events (Shen et al., 2021). Plasma exposure accounted for only about 0.186% of the total systemic exposure, indicating a strong tissue affinity for ammoxetine, potentially resulting in tissue plasma redistribution and sustaining plasma drug concentration over time (Luckenbill et al., 2008).

## Conclusions

The novelty of this project lies in the establishment of the first *in vivo* analysis method for ammoxetine in rats and dogs. We also present the pharmacokinetic parameters of the compound in rats and beagle dogs, along with preliminary speculations and identifications of its metabolic transformation products in rats. In addition, we employed *in vitro* and *in silico* methods to investigate its metabolic characteristics, including metabolic phenotype, metabolic enzyme inhibition, and BBB permeability. These comprehensive findings provide essential experimental data for a thorough understanding of the compound’s pharmacological and toxicological properties. Furthermore, this study strongly supports the development and registration of ammoxetine as a candidate compound, serving as a valuable reference for the design of future clinical evaluations.

## Data Availability

The original contributions presented in the study are included in the article/Supplementary Material, further inquiries can be directed to the corresponding authors.
