# Supplementary material for: In vitro, in vivo, and in silico approaches for evaluating the preclinical DMPK profiles of ammoxetine, a novel chiral serotonin and norepinephrine reuptake inhibitor
Source: Front Pharmacol. 2024 Nov 7;15:1486856. doi: 10.3389/fphar.2024.1486856 (PMC11579541; doi:10.3389/fphar.2024.1486856)
Supplement: Supplementary file 1 [file DataSheet2.pdf]

1. **Development and validation of an LC-MS/MS method for the quantification of ammoxetine in rat plasma**

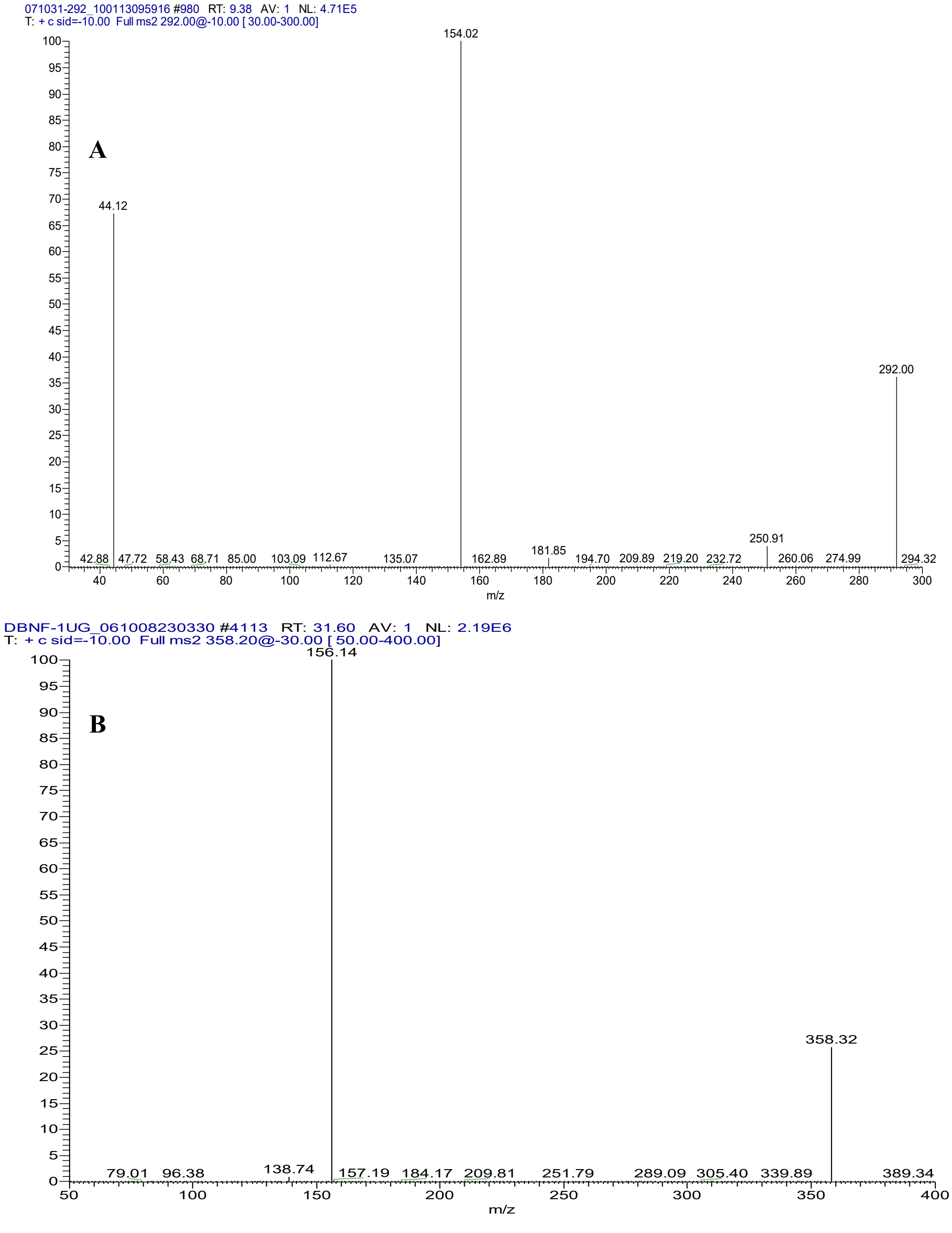

Fig. 1 MS/MS spectrum of $[M+H]^+$ ion of ammoxetine (*m/z* 292, A) and L-phencynonate (IS) (*m/z* 358, B)

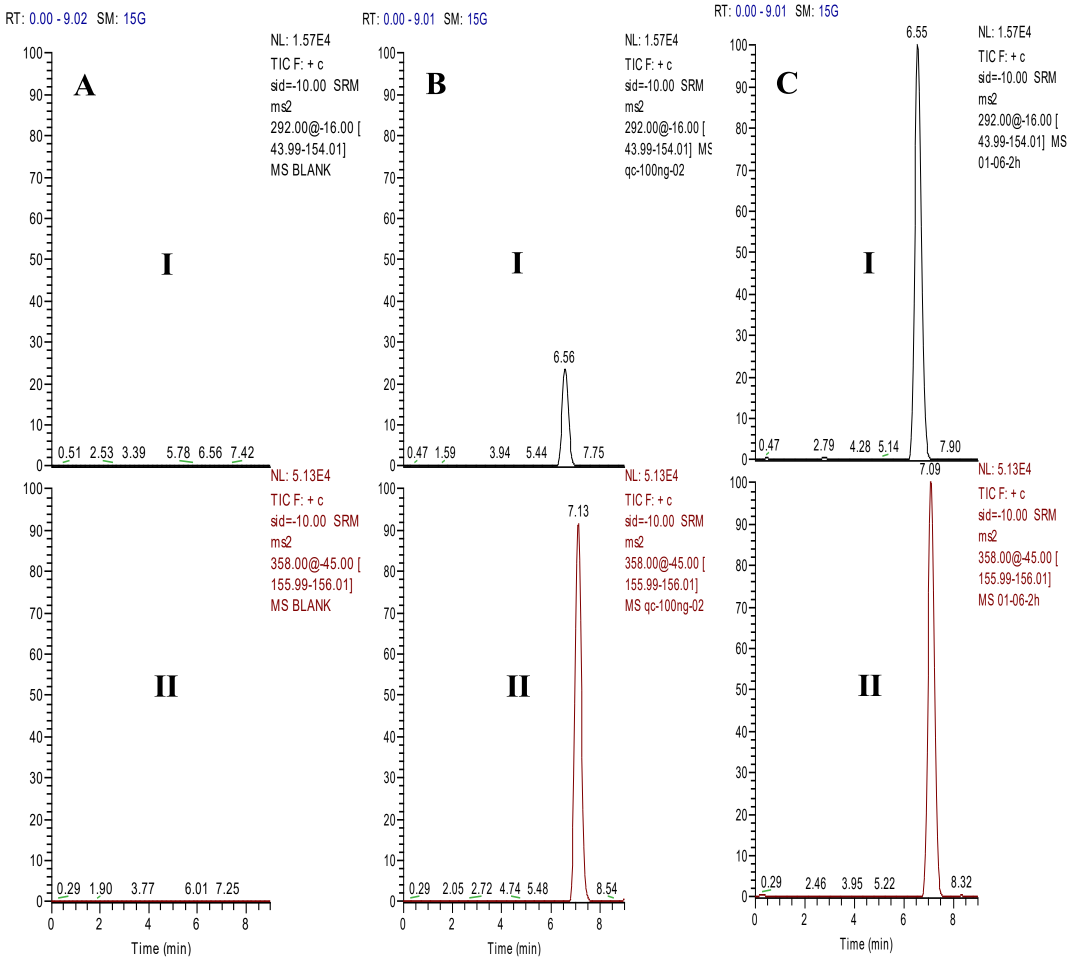

Fig.2 Chromatograms of rat plasma

(A) a blank rat plasma, (B) a blank rat plasma spiked with ammoxetine (100 ng/mL) and internal standard (50 ng/mL), (C) a rat plasma sample obtained 2h after oral administration of a 20 mg/kg dose of ammoxetine; (I) ammoxetine (II) internal standard

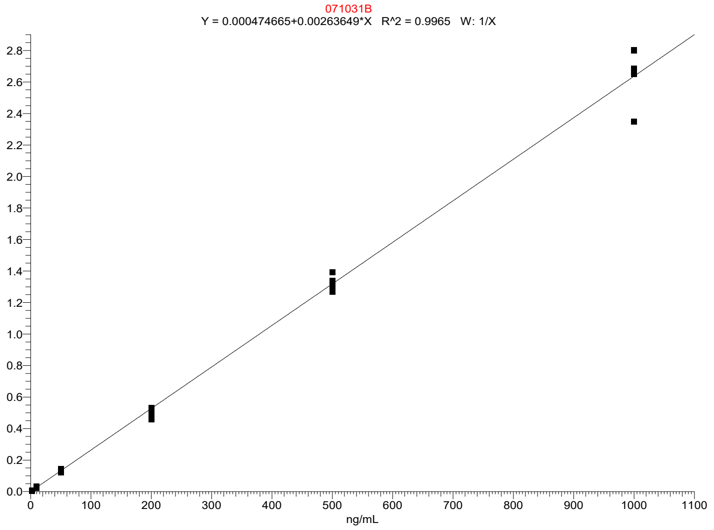

Fig.3 The standard curve of ammoxetine in rat plasma (n=5) with a linear range of 2–1000 ng/mL and a limit of quantification of 2 ng/mL

Tab.1 Precision and accuracy of the assay method for ammoxetine in rat plasma

Added /(ng/mL)	Intra-day precision and accuracy (n=5)			Inter-day precision and accuracy (n=15)		
	Found/ (ng/mL)	Accuracy/%	RSD/%	Found/ (ng/mL)	Accuracy/%	RSD/%
5	4.92±0.50	98.48±9.95	10.10	4.99±0.58	99.8±11.53	11.54
100	103.86±6.94	103.86±6.94	6.68	98.97±6.61	98.97±6.61	6.67
800	849.17±51.87	106.15±6.48	6.11	815.96±64.26	102±8.03	7.88

Tab.2 Extraction recoveries and matrix effects of ammoxetine in rat plasma (mean±SD, n=5)

Added /(ng/mL)	Extraction recovery (%)	Matrix effect (%)
5	95.61±6.05	98.05±3.22
100	102.52±1.88	101.83±8.69
800	95.92±5.48	93.65±4.80

Tab.3 Stability of ammoxetine in rat plasma (mean±SD, n=3)

Added/(ng/mL)	Measured (%)		
	Room temperature for 4h	-30℃ for 15d	In auto-sampler for 24h
5	94.00±6.00	106.00±2.00	114.00±10.00
100	108.90±7.40	105.90±8.30	111.20±5.90
800	114.33±0.73	111.74±2.73	106.49±0.78

2. **Development and validation of an LC-MS/MS method for the quantification of ammoxetine in beagle dog plasma**

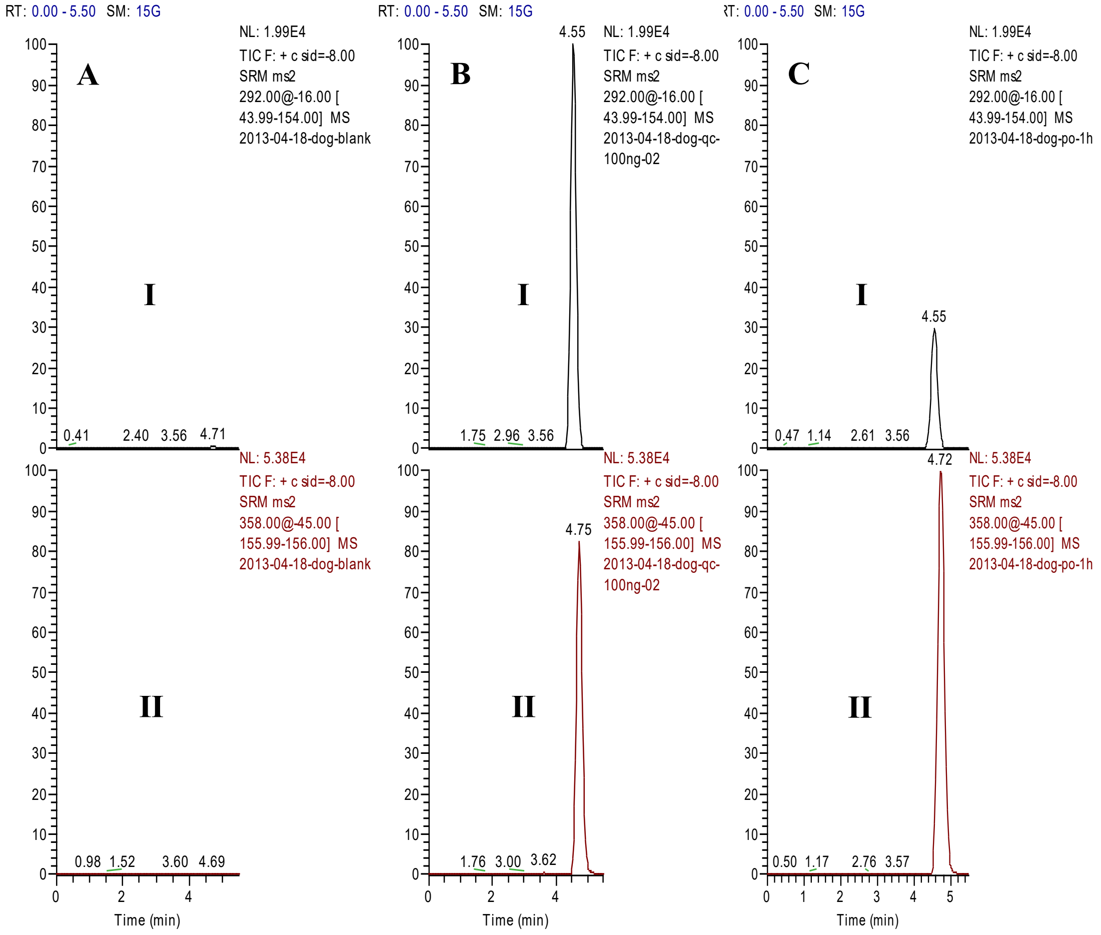

Fig.4 Chromatograms of dog plasma

(A) a blank dog plasma, (B) a blank dog plasma spiked with ammoxetine (100 ng/mL) and internal standard (50 ng/mL), (C) a dog plasma sample obtained 1h after oral administration of a 2 mg/kg dose of ammoxetine; (I) ammoxetine (II) internal standard

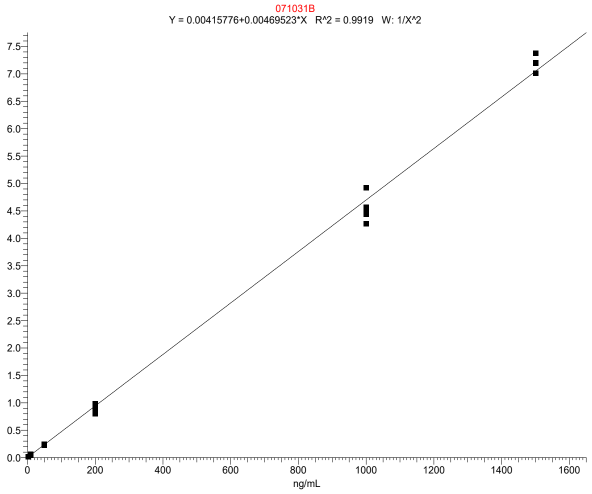

Fig.5 The standard curve of ammoxetine in dog plasma (n=5) with a linear range of 2–1500 ng/mL and a limit of quantification of 2 ng/mL

Tab.4 Precision and accuracy of the assay method for ammoxetine in dog plasma

Added	Antra-day precision and accuracy (n=5)			Inter-day precision and accuracy (n=15)		
/(ng/mL)	Found/ (ng/mL)	Accuracy/%	RSD/%	Found/ (ng/mL)	Accuracy/%	RSD/%
5	5.19±0.33	103.80±6.55	6.31	5.14±0.50	99.6±11.78	9.63
100	104.41±9.30	104.41±9.30	8.91	105.65±7.29	101.70±7.39	6.90
1200	1196.93±107.11	99.74±8.93	8.95	1197.88±100.83	102.85±12.45	8.42

Tab.5 Extraction recoveries and matrix effects of ammoxetine in dog plasma (mean±SD, n=5)

Added /(ng/mL)	Extraction recovery (%)	Matrix effect (%)
5	100.13±7.23	98.10±10.62
100	100.97±4.84	94.98±5.82
1200	97.10±3.26	93.92±2.04

Tab.6 Stability of ammoxetine in dog plasma (mean±SD, n=5)

Found (ng/mL)	Standing Time	Accuracy/%
5	0d (-30℃)	91.60±7.22
	15d (-30℃)	97.58±13.78
	25d (-30℃)	97.61±9.77
	Room temperature for 4h	99.00±11.24
	Repeated freezing and thawing for 3 times	96.59±4.01
	In auto-sampler for 24h	96.64±4.72
100	0d (-30℃)	100.10±1.62
	15d (-30℃)	98.63±4.19
	25d (-30℃)	101.68±6.57
	Room temperature for 4h	101.87±8.27
	Repeated freezing and thawing for 3 times	98.25±6.55
	In auto-sampler for 24h	99.51±7.50
1200	0d (-30℃)	92.06±3.06
	15d (-30℃)	92.48±4.64
	25d (-30℃)	93.96±3.46
	Room temperature for 4h	93.89±4.32
	Repeated freezing and thawing for 3 times	92.01±6.32
	In auto-sampler for 24h	92.32±4.25

3. **Development and validation of LC-MS/MS methods for the quantification of ammoxetine in rat tissues, urine, feces, and bile**

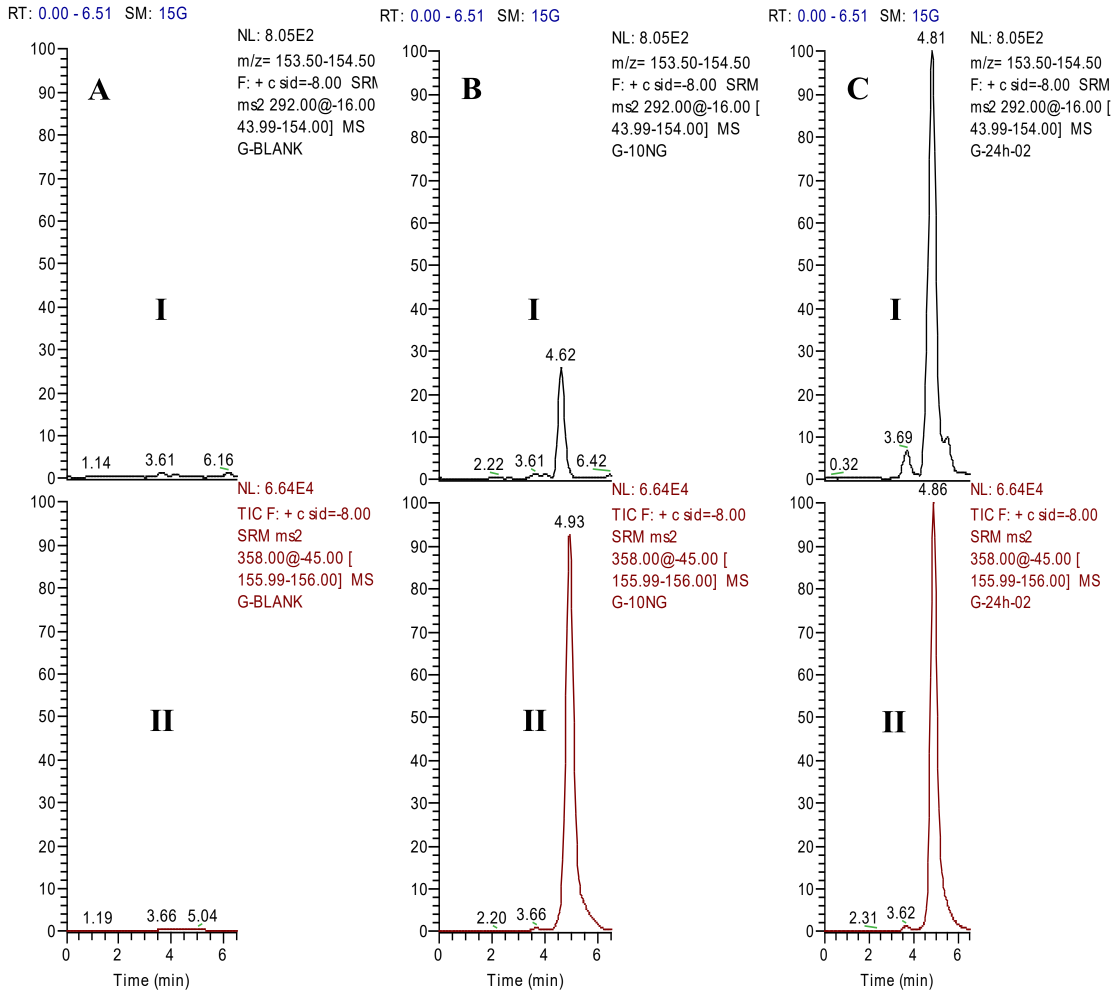

Fig.6 Chromatograms of liver homogenate of rat

(A) a blank liver homogenate of rat, (B) a blank liver homogenate of rat spiked with ammoxetine (10 ng/mL) and internal standard (50 ng/mL), (C) a liver homogenate sample of rat obtained 24h after oral administration of a 20 mg/kg dose of ammoxetine; (I) ammoxetine (II) internal standard

Tab.7 The standard curve of ammoxetine in rat plasma and tissue homogenates

Sample	Regression equation	r^2	Linear range (ng/mL)	Limit of quantification (ng/mL)
Blood	Y = -5.736e-4+4.13e-4×X	0.9945	2–1500	2
Heart	Y = -1.56e-4+2.80e-4×X	0.9981	2–1500	2
Liver	Y = -2.87e-4+2.52e-4×X	0.9986	2–1500	2
Spleen	Y = -4.548e-4+2.80e-4×X	0.9955	2–1500	2
Lung	Y = -1.74e-4+2.36e-4×X	0.9969	2–1500	2
Kidney	Y = -1.76e-4+3.32e-4×X	0.9979	2–1500	2
Brain	Y = 6.49e-5+2.01e-4×X	0.9982	2–1500	2
Intestine	Y = 5.50e-5+3.49e-4×X	0.9943	2–1500	2
Stomach	Y = -3.16e-4+2.28e-4×X	0.9934	2–1500	2
Testis	Y = -4.83e-4+3.54e-4×X	0.9976	2–1500	2
Fat	Y = -4.55e-4+4.36e-4×X	0.9948	2–1500	2
Muscle	Y = -2.90e-4+2.90e-4×X	0.9993	2–1500	2

Tab.8 The standard curve of ammoxetine in urine, feces and bile

Sample	Regression equation	r^2	Linear range (ng/mL)	Limit of quantification (ng/mL)
Urine	Y = 0.011860+0.008805×X	0.9921	2–1000	2
Feces	Y = 0.029786+0.006446×X	0.9979	2–1000	2
Bile	Y = -0.006326+0.005752×X	0.9969	2–1000	2

Tab.9 Extraction recoveries and matrix effects of ammoxetine in different tissue homogenates of rat (n=5)

Tissue	Added (ng/mL)	Extraction recoveries (%)		Matrix effects (%)	
		Mean±SD	RSD	Mean±SD	RSD
Heart	5	110.58±14.58	13.19	84.02±9.45	11.25
	100	97.81±5.18	5.30	97.91±6.52	6.66
	1000	98.91±2.50	2.53	98.70±1.24	1.26
Liver	5	109.93±18.53	16.86	103.10±20.374	19.76
	100	97.58±4.94	5.06	102.68±4.49	4.37
	1000	94.96±3.81	4.01	93.87±1.44	1.53
Spleen	5	123.73±11.39	9.21	73.76±13.84	18.76
	100	101.59±6.92	6.81	104.16±7.01	6.73
	1000	91.79±1.77	1.93	95.62±3.43	3.59
Lung	5	100.01±12.61	12.61	95.97±5.77	6.01
	100	94.75±7.60	8.02	103.65±5.97	5.76
	1000	93.84±1.26	1.34	100.27±3.75	3.74
Kidney	5	92.35±6.56	7.10	149.02±17.99	12.07
	100	99.60±5.80	5.82	100.32±4.40	4.39
	1000	98.71±3.01	3.05	96.20±3.75	3.90
Brain	5	114.23±17.13	14.99	90.68±5.40	5.96
	100	97.71±6.36	6.51	100.33±9.27	9.24
	1000	102.90±5.21	5.06	95.32±4.18	4.39
Intestine	5	88.34±8.13	9.20	90.95±12.69	13.95
	100	102.96±7.30	7.09	100.33±3.01	3.00
	1000	97.97±5.23	5.34	98.35±4.12	4.19
Stomach	5	98.97±11.45	11.57	131.77±25.79	19.57
	100	94.50±5.03	5.32	106.15±11.07	10.43
	1000	93.59±3.17	3.39	106.54±2.49	2.34
Testis	5	99.10±16.57	16.72	87.87±6.54	7.44
	100	102.59±4.52	4.41	100.81±6.39	6.34
	1000	98.77±4.20	4.25	102.71±5.99	5.83
Fat	5	93.44±10.08	10.79	208.15±8.67	4.17
	100	105.06±5.49	5.23	110.51±7.98	7.22
	1000	89.21±11.50	12.89	92.21±2.66	2.88
Muscle	5	99.25±13.83	13.93	89.23±9.29	10.41
	100	93.98±5.18	5.51	91.73±13.24	14.43
	1000	94.08±4.02	4.27	103.10±3.54	3.43

Tab.10 Extraction recoveries and matrix effects of ammoxetine in urine, feces and bile (n=3)

Sample	Added (ng/mL)	Extraction recovery		Matrix effect	
		Mean±SD	RSD (%)	Mean±SD	RSD (%)
	5	86.22±1.60	1.85	98.33±19.33	19.65
Urine	100	92.04±3.24	3.48	88.43±2.80	3.17
	800	94.99±4.71	4.96	100.14±6.44	6.43
	5	109.13±19.11	17.51	101.31±7.32	7.22
Feces	100	110.30±7.49	6.79	90.69±11.23	12.39
	800	98.51±5.66	5.74	98.52±8.04	8.16
	5	87.48±12.84	14.68	43.25±8.30	19.19
Bile	100	103.49±10.29	9.94	49.31±1.15	2.34
	800	106.26±10.35	9.74	48.36±3.38	6.98

4. Development of an LC-MS/MS method for quantifying each metabolite of a CYP-specific substrate in a CYP inhibition experiment.

Tab.11 The standard curve of each metabolite of a CYP-specific substrate

Analyte	Regression equation	*r*	Linear range (ng/mL)	Limit of quantification (ng/mL)
4-Hydroxytolbutamide	$Y = 1.72e^{+003}+3.6e^{+003}\times X$	0.9988	0.25–500	0.25
4-Hydroxymephenytoin	$Y = 29.3+144\times X$	0.9992	0.25–500	0.25
Acetaminophen	$Y = 0.459+1.03\times X$	0.9900	0.1–200	0.1
Hydroxybupropion	$Y = 0.131+0.942\times X$	0.9921	0.15–300	0.15
6-hydroxychlorzoxazone	$Y = 577+579\times X$	0.9934	0.15–300	0.15
1'-hydroxymidazolam	$Y = 0.148+0.162\times X$	0.9934	0.025–50	0.025
6β-hydroxytestosterone	$Y = 0.0146+0.0813\times X$	0.9912	0.2–400	0.2
Dextrorphan	$Y = 0.12+1.64\times X$	0.9947	0.025–50	0.025
7-hydroxycoumarin	$Y = 986+2.26e^{+003}\times X$	0.9931	0.025–50	0.025
Desethylamodiaquine	$Y = 0.599+2.37\times X$	0.9950	0.025–50	0.025
