# Supplementary material for: In vitro, in vivo, and in silico approaches for evaluating the preclinical DMPK profiles of ammoxetine, a novel chiral serotonin and norepinephrine reuptake inhibitor
Source: Front Pharmacol. 2024 Nov 7;15:1486856. doi: 10.3389/fphar.2024.1486856 (PMC11579541; doi:10.3389/fphar.2024.1486856)
Supplement: Supplementary file 2 [file Table1.pdf]

Tab. S1 Cytotoxicity test results of ammoxetine by using MTT assay (n=6)

Concentration of ammoxetine (μg/mL)	Absorbance value (A)	RGR (%)
0	0.426±0.038	100.00±8.90
$1.28\times e^{-3}$	0.482±0.096	113.13±22.65
$6.40\times e^{-3}$	0.489±0.063	114.88±14.68
$3.20\times e^{-2}$	0.441±0.060	103.57±14.18
0.16	0.440±0.040	103.48±9.43
0.80	0.437±0.077	102.55±18.09
4	0.441±0.089	103.59±20.82
20	0.380±0.055	89.16±12.89
100	0.236±0.034	55.53±7.87
500	0.104±0.010	24.49±2.36

Tab. S2 P_{app} values after 2 h of incubation on the bidirectional permeability measurements for ammoxetine in the MDCK-MDR1 cell line (n=3)

Concentration of	P_{app} ($\times10^{-6}$cm/s)			E_R [$P_{app(A\rightarrow B)}/P_{app(B\rightarrow A)}$]
ammoxetine (μg/mL)	A→B	A→B (with verapamil)	B→A	
0.2	15.827±2.833	27.224±6.435	26.469±5.299	1.673
1	6.605±0.555	28.815±1.892	11.517±2.929	1.744
5	13.250±1.264	29.858±3.291	25.192±3.492	1.901

Note. apical to basolateral: A→B; basolateral to apical: B→A

Tab. S3 The transformation ratio of ammoxetine in recombinant CYP isoforms (n=5)

Group	The residuary concentration of ammoxetine					Mean±SD	Transformation ratio%
	1	2	3	4	5		
Zero time control	6.90	6.93	6.51	5.94	7.99	6.85±0.75	0
1A2	5.71	5.76	5.97	6.08	5.94	5.89±0.16	14.04±2.29
2C19	5.03	5.21	4.99	4.90	5.15	5.05±0.13*	26.27±1.85
2C9	5.43	6.40	6.45	5.48	6.06	5.96±0.49	13.02±7.14
2D6	5.73	5.96	5.81	6.07	5.87	5.89±0.13	14.09±1.89
3A4	4.88	5.22	5.10	5.11	5.56	5.18±0.25*	24.49±3.65

*P<0.05 *vs.* zero time control group
