# Supplementary material for: In vitro, in vivo, and in silico approaches for evaluating the preclinical DMPK profiles of ammoxetine, a novel chiral serotonin and norepinephrine reuptake inhibitor
Source: Front Pharmacol. 2024 Nov 7;15:1486856. doi: 10.3389/fphar.2024.1486856 (PMC11579541; doi:10.3389/fphar.2024.1486856)
Supplement: Supplementary file 3 [file DataSheet1.pdf]

In vivo study

Configurational stability study.

Instrument: Agilent 1100 LC/MSD VL LC-MS system

Column: CHIRALPAK® AD-H column (4.6 mm×250 mm, 5 μm)

Mobile phase: n-hexane: isopropanol: diethylamine = 94.8: 5: 0.2 (*v*/*v*/*v*)

Flow rate: 1 mL/min

UV detection wavelength: 275 nm

Injection volume: 10 μL

Column temperature: room temperature

PK study in rats.

Instrument: Finnigan TSQ Quantum LC-MS-MS equipped with an electrospray ionization (ESI) source

1) Chromatography conditions:

Column: Thermo Fisher C_{18} column (2.1 mm×50 mm, 5 μm)

Mobile phase: water (A) and acetonitrile (C), both contain 0.1 % formic acid

Gradient elution: 0.0 ~ 3.0 min, 90 % ~ 60 % A; 3.0 ~ 4.0 min, 60 % ~ 5 % A; 4.1 ~ 9.0 min, 90 % A

Flow rate: 0.3 mL/min

Injection volume: 10 μL

Column temperature: room temperature

2) MS conditions:

Spray voltage: 4.5 kV

Nitrogen sheath gas flow: 25 psi

Nitrogen auxiliary gas flow: 10 psi

Argon collision gas pressure: 1.0 mTorr

Heated capillary temperature: 270 °C

Source collision induced dissociation (CID): 10 V

Scan mode: positive ionization mode with selected reaction monitoring (SRM) mode of *m/z* 292→154, 44 for ammoxetine, and *m/z* 358→156 for internal standard (IS, L-phencynonate), respectively.

Collision energy: 16 eV for ammoxetine and 45 eV for IS, respectively.

PK study in dogs.

Instrument: Finnigan TSQ Quantum LC-MS-MS equipped with an ESI source

1) Chromatography conditions:

Mobile phase: water (A) and acetonitrile (C), both contain 0.1 % formic acid

Gradient elution: 0.00 ~ 0.60 min, 95 % ~ 20 % A; 0.60 ~ 1.50 min, 20 % ~ 5 % A; 1.51 ~ 5.50 min, 95 % A

The other conditions were identical to those described in the PK study in rats.

2) MS conditions:

Spray voltage: 4.8 kV

Nitrogen sheath gas flow: 40 psi

Nitrogen auxiliary gas flow: 5 psi

Heated capillary temperature: 300 °C

Source CID: 8 V

The other conditions were identical to those described in the PK study in rats.

Tissue distribution study in rats.

Instrument: Finnigan TSQ Quantum LC-MS-MS equipped with an ESI source

1) Chromatography conditions:

Mobile phase: water (A) and acetonitrile (C), both contain 0.1 % formic acid

Gradient elution: 0.00 ~ 0.60 min, 95 % ~ 25 % A; 0.60 ~ 1.50 min, 25 % ~ 5 % A; 1.51 ~ 6.50 min, 95 % A

The other conditions were identical to those described in the PK study in dogs.

2) MS conditions:

Scan mode: positive ionization mode with SRM mode of *m/z* 292→154 for ammoxetine, and *m/z* 358→156 for IS (L-phencynonate), respectively.

The other conditions were identical to those described in the PK study in dogs.

Excretion study in rats.

Instrument: Finnigan TSQ Quantum LC-MS-MS equipped with an ESI source

The conditions were identical to those described in the PK study in dogs.

Initial identification of major metabolites

Instrument: Finnigan TSQ Quantum LC-MS-MS equipped with an ESI source

1) Chromatography conditions:

Column: Thermo Fisher C_{18} column (2.1 mm×100 mm, 5 μm); Thermo Fisher C_{18} column (2.1 mm×50 mm, 5 μm) for neutral-loss scan

Mobile phase: water (A) and acetonitrile (C), both contain 0.1 % formic acid

Gradient elution: 0 ~ 10 min, 80 % ~ 50 % A; 10 ~ 20 min, 50 % ~ 25 % A; 20 ~ 25 min, 25 % ~ 10 % A; 25 ~ 30 min, 10 % ~ 25 % A; 30 ~ 35 min, 25 % ~ 50 % A; 35 ~ 40 min, 50 % ~ 80 % A; 40 ~ 60 min, 80 % A

Flow rate: 0.2 mL/min

The other conditions were identical to those described in the PK study in dogs.

2) MS conditions:

Spray voltage: 4.5 kV

Nitrogen sheath gas flow: 25 psi

Nitrogen auxiliary gas flow: 10 psi

Argon collision gas pressure: 1.0 mTorr

Heated capillary temperature: 270 °C

Source CID: 10 V

Scan mode: positive/negative ionization modes with full scan MS (scan range of *m/z* 150 ~ 650); positive ionization mode with full scan MS/MS (collision voltage: 16 V; parent ions: *m/z* 292, *m/z* 280, and *m/z* 456, and the corresponding scan ranges of *m/z* 30 ~ 300, *m/z* 30 ~ 300, and *m/z* 30 ~ 500, respectively); positive/negative ionization modes with neutral-loss scan (collision voltage: 25 V; the *m/z* of neutral loss fragment: 176; scan range of *m/z* 150 ~ 800).

In vitro study

Bidirectional permeability measurements in MDCK-MDR1 transport study.

Instrument: Finnigan TSQ Quantum LC-MS-MS equipped with an ESI source

The conditions were identical to those described in the PK study in dogs.

Hepatic microsome stability assay.

Instrument: Finnigan TSQ Quantum LC-MS-MS equipped with an ESI source

Mobile phase: water (A) and acetonitrile (C), both contain 0.1 % formic acid

Gradient elution for ammoxetine: 0 ~ 2.0 min, 90 % ~ 50 % A; 2.0 ~ 2.5 min, 50 % ~ 90 % A; 2.5 ~ 7.5 min, 90 % A

Isocratic elution for verapamil: water: acetonitrile = 60: 40 (*v*/*v*), both contain 0.1 % formic acid

The other chromatography and MS conditions were identical to those described in the PK study in dogs.

Phenotyping of recombinant human CYP isoenzyme.

Instrument: Finnigan TSQ Quantum LC-MS-MS equipped with an ESI source

Mobile phase: water (A) and acetonitrile (C), both contain 0.1 % formic acid

Gradient elution: 0 ~ 3.0 min, 90 % ~ 60 % A; 3.0 ~ 4.0 min, 60 % ~ 5 % A; 4.0 ~ 4.1 min, 5 % ~ 90 % A; 4.1 ~ 9.0 min, 90 % A

The other chromatography and MS conditions were identical to those described in the PK study in rats.

CYP inhibition experiment.

Instrument: AB Sciex API 5000 LC-MS-MS system equipped with a Shimadzu LC-20AD HPLC system and an ESI source

1) Chromatography conditions:

Column: Thermo Fisher C_{18} column (2.1 mm×100 mm, 5 μm)

Mobile phase: water (A, containing 1 mmol/L ammonium acetate and 0.02 % formic acid) and acetonitrile (B)

Gradient elution: 0 ~ 0.5 min, 5 % B; 0.5 ~ 3 min, 5 % ~ 95 % B; 3 ~ 3.1 min, 95 % ~ 5 % B; 3.1 ~ 6 min, 5 % B

Flow rate: 0.4 mL/min

Injection volume: 4 μL

Column temperature: 40 °C

2) MS conditions:

IonSpray voltage: 4.5 kV

Temperature: 550 °C

Curtain gas: 30 L/h

Collision gas: 12 L/min

Ion source gas 1: 40 L/min

Ion source gas 2: 60 L/min

Scan mode: positive/negative ionization modes with multiple reaction monitoring (MRM), using a 5 ms dwell time.

For IS and metabolites from probe substrate, their scan modes, retention times (t_R), and the other MS parameters, including declustering potential (DP), entrance potential (EP), collision energy (CE), and collision cell exit potential (CXP), were listed in the following Table:

CYPs	Substrates	Analyte	Transition (*m/z*)	Mode	CE (V)	DP (V)	EP (V)	CXP (V)	t_R (min)
2C9	Tolbutamide	4-Hydroxytolbutamide	285.0→ 186.2	-	-25	-120	-10	-12	2.77
2C19	S-mephenytoin	4-Hydroxymephenytoin	232.9→ 161.1	-	-25	-120	-10	-12	2.56
1A2	Phenacetin	Acetaminophen	152.0→ 110.2	+	25	120	10	12	1.07
2B6	Bupropion	Hydroxybupropion	256.0→ 131.2	+	38	120	10	12	2.55
2E1	Chlorzoxazone	6-hydroxychlorzoxazone	183.5→ 120.0	-	-25	-120	-10	-12	2.32
3A4	Midazolam	1’-hydroxymidazolam	342.0→ 324.3	+	30	120	10	12	2.96
3A4	Testosterone	6β-hydroxytestosterone	305.0→ 269.4	+	22	120	10	12	2.79
2D6	Dextromethorphan	Dextrorphan	258.1→ 199.2	+	40	120	10	12	2.48
2A6	Coumarin	7-hydroxycoumarin	160.4→ 133.3	-	-25	-120	-10	-12	2.26
2C8	Amodiaquine	Desethylamodiaquine	328.0→ 283.1	+	25	120	10	12	2.24
		Dexmedetomidine (IS)	201.0→ 94.9	+	55	100	10	12	2.71

Plasma protein binding assay.

Instrument: Finnigan TSQ Quantum LC-MS-MS equipped with an ESI source

The conditions were identical to those described in the PK study in dogs.
