# Supplementary material for: In vitro, in vivo, and in silico approaches for evaluating the preclinical DMPK profiles of ammoxetine, a novel chiral serotonin and norepinephrine reuptake inhibitor
Source: Front Pharmacol. 2024 Nov 7;15:1486856. doi: 10.3389/fphar.2024.1486856 (PMC11579541; doi:10.3389/fphar.2024.1486856)
Supplement: Supplementary file 4 [file Image1.pdf]

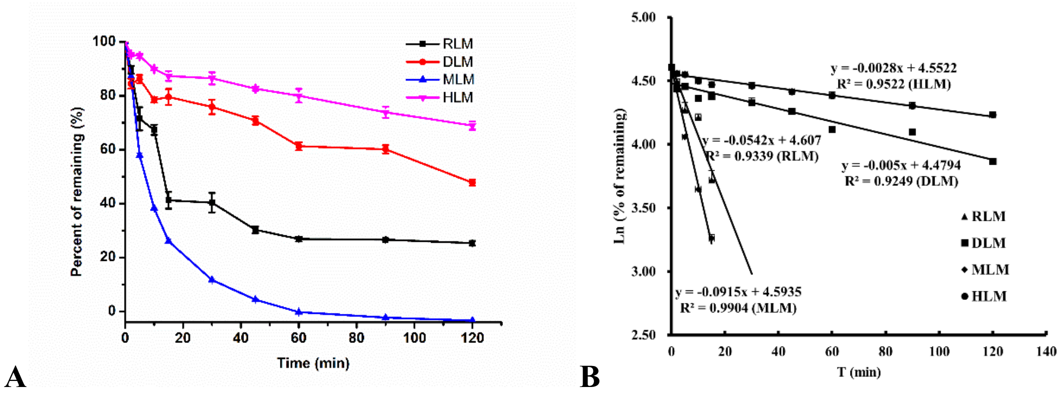

Fig. S1 *In vitro* metabolic kinetics of ammoxetine in human, beagle dog, monkey, and rat liver microsomes (n=3)
